# CAR-T-Cell-Based Cancer Immunotherapies: Potentials, Limitations, and Future Prospects

**DOI:** 10.3390/jcm13113202

**Published:** 2024-05-29

**Authors:** Mahmood S. Choudhery, Taqdees Arif, Ruhma Mahmood, David T. Harris

**Affiliations:** 1 Department of Human Genetics & Molecular Biology, University of Health Sciences, Lahore 54600, Pakistan; taqdeesarif01@gmail.com; 2 Jinnah Hospital, Allama Iqbal Medical College, Lahore 54700, Pakistan; ruhma_mahmood@yahoo.com; 3 Department of Immunobiology, College of Medicine, University of Arizona Health Sciences Biorepository, The University of Arizona, Tucson, AZ 85724-5221, USA; davidh@email.arizona.edu

**Keywords:** CAR-T therapy, CRISPR-Cas9, artificial intelligence, Boolean-gated CARs

## Abstract

Cancer encompasses various elements occurring at the cellular and genetic levels, necessitating an immunotherapy capable of efficiently addressing both aspects. T cells can combat cancer cells by specifically recognizing antigens on them. This innate capability of T cells has been used to develop cellular immunotherapies, but most of them can only target antigens through major histocompatibility complexes (MHCs). New gene-editing techniques such as clustered regularly interspaced short palindromic repeat (CRISPR)-associated protein 9 (CRISPR-cas9) can precisely edit the DNA sequences. CRISPR-cas9 has made it possible to generate genetically engineered chimeric antigen receptors (CARs) that can overcome the problems associated with old immunotherapies. In chimeric antigen receptor T (CAR-T) cell therapy, the patient’s T cells are isolated and genetically modified to exhibit synthetic CAR(s). CAR-T cell treatment has shown remarkably positive clinical outcomes in cancers of various types. Nevertheless, there are various challenges that reduce CAR-T effectiveness in solid tumors. It is required to address these challenges in order to make CAR-T cell therapy a better and safer option. Combining CAR-T treatment with other immunotherapies that target multiple antigens has shown positive outcomes. Moreover, recently generated Boolean logic-gated advanced CARs along with artificial intelligence has expanded its potential to treat solid tumors in addition to blood cancers. This review aims to describe the structure, types, and various methods used to develop CAR-T cells. The clinical applications of CAR-T cells in hematological malignancies and solid tumours have been described in detail. In addition, this discussion has addressed the limitations associated with CAR-T cells, explored potential strategies to mitigate CAR-T-related toxicities, and delved into future perspectives.

## 1. Background

Cancer is a complex group of disorders described by uncontrolled cell growth and metastasis, affecting millions of people every year. Its epidemiology varies across regions and is influenced by factors such as genetics, epigenetics, lifestyle choices, and environmental exposures contributing to its diverse impact worldwide. The origin of abnormal cell growth in cancer requires tailored approaches involving chemotherapy, surgery, and radiation therapy. However, these methods employed to kill cancer cells often lead to significant adverse effects and increase the chances of cancer recurrence. Consequently, alternative, long-term approaches are required to effectively treat cancer. Immunotherapy is an alternative treatment strategy against cancer. Immunotherapy improves the patient’s immune system by altering the immune responses, thereby making it possible to find and attack the cancer cells [1].

The first demonstration that the immune system of a patient can stop tumor growth was presented in 1957 by Burnet. Since then, a large number of studies have supported this notion and emphasized the involvement of immune cells as positive prognostic candidates for various types of cancers [2,3,4]. The growing understanding of immune cells and their potential enabled the development of new biological treatments with positive therapeutic results. These treatments make the patient immune system act like a living drug to find and destroy cancer cells. Immune cells can identify and eradicate the damaged or infected cells in addition to those cells that have become cancerous. Killer T cells are especially intriguing against cancer, owing to their ability to bind to antigens on tumor cells. Cellular immunotherapies take the benefit of this natural ability of T cells and can be implemented in different ways as a cancer treatment [3]. Previously, various types of immunotherapies, such as monoclonal antibody treatment, immune checkpoint inhibitors, cytokine therapy, and adoptive cell therapies (ACTs), have been successfully employed to treat cancers. ACT is of particular interest as it uses the patient’s own immune cells to combat cancer. Tumor-infiltrating lymphocyte (TIL) therapy and chimeric antigen receptor (CAR) T cell therapy are the two main types of ACT [5]. In TIL therapy, T cells that have already infiltrated a patient’s tumors are expanded in vitro and are activated to obtain a sufficient number. These activated cells are then re-infused into the patient, where they can recognize and destroy the cancerous cells [3].

The mere existence of T cells in patients does not always guarantee the elimination of cancer cells because not all patients have T cells that have invaded tumors (e.g., in TILs). Similarly, T cells may not be capable of activating and expanding in adequate quantities. For such patients, engineered T cell receptor (TCR) therapy is advised in which T cells are not only expanded and activated but also equipped with a new T cell receptor. The new receptor allows the T cells to target particular cancer antigens. Both the TIL and TCR therapies, however, can only target and eliminate cancer cells that present antigens through the MHC. To overcome this limitation, those engineered T cells that are not restricted by the MHC are required. Recently, CRISPR-Cas9 (clustered regularly interspaced short palindromic repeats (CRISPR)-associated protein 9), a powerful gene-editing technique, has enabled investigators to generate CAR-T cells, a type of ACT that is safe, potent, and specific for cancer treatment. It is pertinent to note that gene-editing techniques have the potential to generate reproducibly effective and robust universal cell products for immediate cancer treatment. CAR-T cell therapy employs genetically modified T cells that can bind to a specific protein on the cancerous cells. This binding allows the T cells to attack the cancer cells more precisely and eliminate the problems associated with older immunotherapies. In CAR-T therapy, patient T cells are isolated from peripheral blood and genetically modified by adding a synthetic receptor called a CAR. CAR-T cells are then cultured and amplified in vitro before being reinfused into patients as a cancer treatment. Genetically modified CAR-T cells then identify and eradicate cells that express a tumor-associated antigen. However, unlike most of the older immunotherapies, CARs are not restricted by the MHC because CARs are constructed from the antibody-binding region, which does not require antigen presentation by the MHC. This strategy can make more cancer cells susceptible to attacks, thereby making it more effective against cancer [6].

CAR-T-based therapy has been successfully employed to treat certain hematological malignancies, such as leukemia and lymphoma. Significant and sustained remissions of cancer in patients because of CAR-T therapy are considered a remarkable advancement in the development of personalized cancer treatment. Its success had led to FDA approval for treating blood cancers. Two CAR-T-based therapies, i.e., axicabtagene ciloleucel and tisagenlecleucel, target CD19 and have been approved by the FDA in 2017 to treat non-Hodgkin’s lymphoma (NHLs) and relapsed acute lymphoblastic leukemia (ALL), respectively. The FDA also approved Brexucabtagene autoleucel, a new CAR-T therapy for adult patients with relapsed mantle cell lymphoma (MCL). Likewise, in 2021, lisocabtagene maraleucel was approved for diffuse large B cell lymphoma (DLBCL) [7]. Moreover, the FDA recently approved the first clinical trial of IMPT-314, an advanced logic-gated CAR-T treatment for patients with B cell lymphoma. This study was launched early in 2023 and is expected to be completed by 2029 [8].

Although CAR-T cell treatment has been successful and groundbreaking outcomes have been achieved for hematological cancer treatment, there are certain challenges and limitations. Current research is focusing on solving these issues and making this treatment error-free [7]. Furthermore, Boolean logic-gated advanced CARs and artificial intelligence (AI) have offered insights and solutions to address the manufacturing challenges of CAR-T cell therapy [9]. This review comprehensively explores current cancer immunotherapies, CARs structure, generations, development methods, clinical applications in different cancers, limitations, and possible solutions. The potential and challenges of using AI in CAR-T cell treatment have also been discussed. The collective effort in research and development strives to enhance CAR-T therapy, making it a more accessible and effective cancer treatment option.

## 2. Introduction to Cancer: Understanding the Complex Landscape of a Pervasive Disease

Cancer results in an aberrant cell growth of cells in the body. The prevalence of cancer differs across geographical regions due to various factors, including epigenetics, genetics, lifestyle choices, and environmental exposures. Cancer development is a multistep process at the cellular level and may involve mutations in the DNA. These mutated cells have the ability to invade, survive, proliferate, and metastasize progressively in the body. Cancer can even begin with a single mutation in a cell that proliferates abnormally. Additional mutations and subsequent selection for rapidly dividing cells lead to the growth and spread of the tumor to other parts of the body [1]. There are over one hundred different kinds of cancer, each with its own characteristics and treatment options. The type of cell that gives rise to the tumor determines its classification. Most cancers are classified into three main groups, including carcinomas, lymphomas or leukemias, and sarcomas. Carcinomas, which make up about 90% of human cancers, are cancer of the cells that line the surfaces of organs and tissues. Lymphomas and leukemias, which comprise about 8% of human cancers, are cancer of cells that fight infections and produce blood cells, respectively. Sarcomas are very rare in humans and affect those organs that provide support to our body, such as cartilage, bone, muscle, and fibrous tissue [10]. Cancers are also classified based on the origin of tissue or organ (e.g., breast, lungs, or kidney) and the specific types of cells. For example, red blood cells and fibroblasts are involved in the development of leukemia and fibroblastoma, respectively. The common treatment methods for cancer include surgery, chemotherapy, and radiation therapy. These methods have adverse side effects and a high risk of recurrence despite effectively reducing and eliminating cancer cells. Hence, alternative long-lasting methods for combating cancers are required. Immunotherapy is an alternative method for combating cancer that modulates the immune response to either directly target the cancer cells or increase the overall immune response [11].

## 3. Current Cancer Immunotherapies

Immunotherapy is one of several cancer treatment options that stimulates the immune response to help the body fight against cancers. Currently available major types of immunotherapies include the application of monoclonal antibodies, checkpoint inhibitors, cytokine therapy, and adoptive cell therapy (ACT) [5,12]. Each type of immunotherapy targets a different type or component of cancer cell or immune system. Immunotherapies may target cancers of various types, such as skin cancer, prostate cancer, bladder cancer, lung cancer, leukemias, lymphomas, and other cancers. Nonetheless, the success rate of immunotherapies varies among different individuals and cancers. It is pertinent to note that these therapies do not work for all types of cancers and therefore all cancer patients do not obtain benefits from them. The success of an immunotherapy is determined by a number of factors, including the level of genetic modification(s) in the cancer cells, previously received treatment, the stage and type of cancer, and the overall health of the immune system of the patient. Studies indicate that immunotherapies can be more effective if combined with the approaches described above (such as surgery, chemotherapy, or radiation therapy). Scientists are continuously exploring new ways to use the potential of the immune system to fight various types of cancers. Several clinical trials have been registered to evaluate the effectiveness of newly developed drugs and combination therapies (www.clinicaltrials.org). In addition, advanced tools have been developed to predict who can benefit from these therapies [5,12,13].

### 3.1. Monoclonal Antibody Therapy

Monoclonal antibody therapy (mAb) is a successful approach to treat specific cancer types, including hematological malignancies and certain solid tumors. In this therapy, laboratory-made antibodies are used to mimic the immune system’s ability to find and kill cancer cells. mAbs are designed to inhibit those factors that stimulate cancer growth or specifically target proteins that are overexpressed on the surface of cancer cells. mAbs are utilized in the following ways to disrupt the development of cancer cells: (1) interfering with the signaling pathways that promote cancer development, (2) delivering chemicals (such as toxins or chemotherapy drugs) or radioactive substances directly to cancer cells to inhibit metastasis to other parts of the body, (3) stimulating the immune system to specifically find and attach to cancer cells and destroy them [5].

mAbs were first produced by hybridomas technology by Kohler and Milstein. The first antibody made, however, was derived from mice, and humans encountered immunological reactions against it. Humanized antibodies were developed later and it was found that they are more compatible with the human immune system. Glycolipids, glycoproteins, cluster differentiation (CD) molecules, vascular targets, carbohydrates, extracellular matrix antigens, stroma, and growth factors are among the targets for humanized antibodies. The first monoclonal antibody for cancer treatment i.e., Rituxan^®^ (rituximab), was approved by the FDA in 1997. It targets the CD20 of B cells and is used for certain types of non-Hodgkin’s lymphoma and chronic lymphocytic leukemia patients. Similarly, blinatumomab (Blincyto) was designed to target CD19 (a protein found on the surface of B cells) and CD3 (a protein found on the surface of T cells). This medication brings the T cells and cancer cells close together and facilitates the destruction of cancer cells. This antibody is primarily prescribed for non-Hodgkin’s lymphoma and B-ALL [14,15]. Monoclonal antibody therapy is a promising cancer treatment option but has some limitations. For example, it may not work for all cancers because all cancers either do not exhibit unique molecules or may become mutated or resistant to it. The effectiveness of mAb therapy varies depending on the patient and cancer type. The availability and wide access are limited as it requires special equipment and facilities to administer the antibodies. In addition, it is prohibitively expensive due to the time-consuming and complicated steps involved in testing and designing the antibodies. Furthermore, depending on the dose and type of mAb used, it may cause certain side effects, such as infections, allergic reactions, organ damage, or inflammation [5,12]. Research in the field of mAb therapy for cancer is ongoing, with efforts to discover new targets, improve treatment efficacy, and minimize side effects.

### 3.2. Immune Checkpoint Inhibitors (ICIs)

Immune checkpoint inhibitors are one of the natural components of the immune system that prevent the immune cells from over-reacting and destroying the body’s healthy cells. The immunological checkpoints are activated when T cells recognize and adhere to surface proteins (known as immune checkpoint proteins) on other cells, including tumor cells. When the checkpoint proteins and their partner proteins bind, T cells receive an “off” signal. This action could prevent the cancer from being eradicated by the immune system (T cells). ICI therapy uses drugs to block the immune checkpoint proteins [12]. PD-1 and CTLA-4 are the two key checkpoints that have been targeted to treat a variety of cancers. For example, PD-1 has shown promising outcomes in the treatment of cutaneous melanoma, Hodgkin’s lymphoma, kidney cancer, lung cancer, and bladder cancer. CTLA-4 prevents aggressive immune responses in lung cancer, kidney cancer, bladder cancer, and skin cancer [16]. Although many types of cancers have been treated with this therapy, one major issue with ICIs is that they can cause immune responses to become uncontrolled and attack normal cells in the body. New alternative cancer therapy approaches are emerging to avoid these negative side effects. One of these approaches is the lymphocyte-activation gene 3 (LAG3), also known as CD223, which is expressed on the surface of activated T cells, regulatory T cells (Tregs), B cells, and natural killer (NK) cells. LAG3 binds with MHC-II molecules on antigen-presenting cells (APCs: dendritic cells and macrophages). When LAG3 binds to MHC-II, it prevents the activation and expansion of T cells. Lag3 is expressed together with the PD-1 marker, so double blockades have many prospects in cancer immunotherapy. Double blockades maintain immune hemostasis and overcome the immunosuppressive roles of the tumor immune microenvironment [17]. In continuation with that, other novel checkpoint inhibitors such as VISTA, B7-H3, PD-1H, CA-170, and TIM-3 have been employed in different clinical studies to treat blood and solid cancers. Although these checkpoint inhibitors have side effects, such as neurological, gastrointestinal, respiratory, and allergic problems, administration of nanoparticles can avoid these side effects. More research and clinical trials are still required to improve the effectiveness and safety of these drugs and to determine the best way to combine it with other treatments [5,18].

### 3.3. Cytokine Therapy

Cytokine therapy includes the use of cytokines, small proteins that affect the immune system. Cytokines play a crucial role in cell signaling and the regulation of immune responses. Cytokines are produced naturally by the body or manufactured in a laboratory and injected into the patient. Cytokines can have a variety of impacts on cancer cells, including immune system activation to combat cancer, stopping the growth of cancer, or directly causing the cancer cells to die. Interferons, interleukins, and tumor necrosis factor are examples of cytokines utilized in cancer therapy. The FDA has approved two cytokines, i.e., IL-2 and IFN- α2b, for the treatment of metastatic melanoma and renal carcinoma. High doses of IFN-α may have numerous benefits for the immune system but can also have side effects on actions such as dendritic cell maturation, apoptosis, and the augmentation of cytotoxic T cell response against tumor cells. IL-2 is an important cytokine that promotes the growth of immune system cells, but it can be toxic. Some new IL-2-based medications are being explored in conjunction with other drugs that block some of the signals that cancers employ to eliminate detection by the immune system. Some other cytokines, such as IL-10, IL-12, IL-15, IL-21, and GM-CSF, boost the immune system and help in fighting the cancer [19,20]. These cytokines have distinct roles in the battle against cancer and the development of new immunotherapies. Despite promising results, cytokine therapy has several limitations, including toxicity, low efficacy, and delivery challenges. Researchers are exploring new strategies to improve cytokines by understanding their interactions and mechanisms in the tumor microenvironment, as well as developing innovative engineering and delivery methods [5].

### 3.4. Adoptive Cell Therapies (ACTs) for Cancers

ACT combats cancer by harnessing the power of the patient’s own immune cells. It involves the isolation, expansion, and transfer of immune cells (usually T cells) into a patient’s body with the aim of treating cancer. The idea of ACT traces back to the mid-20th century, when scientists injected white blood cells from one individual to another. These attempts, however, faced challenges, including graft-versus-host disease (GvHD), a harmful complication where the donor cells assault the recipient’s tissues. The identification and characterization of T cells helped in the development and advancement of ACT. The discovery of T cell subtypes, such as cytotoxic T cells and helper T cells, laid the foundation for understanding cellular immunity. Southam et al. initially demonstrated the potential of ACT in 1966. They investigated the effects of combining autologous T cells with tumor cells as a form of immunotherapy. They observed that, in some patients with advanced cancer, the tumors were reduced following this treatment approach. The authors suggested that the immune response was being stimulated by the combination of T cells and tumor cells, leading to an anti-tumor effect [21]. Overall, the ACT procedure involves isolating T cells from the blood of the patient, boosting and altering the cells in a laboratory, and then reintroducing the cells to the patient to target and eradicate the cancer cells. Hematological malignancies, such as lymphoma and leukemia, have responded well to ACT [22]. However, there are numerous limitations of using ACT for solid tumors. Scientists are developing novel methods to improve the development and administration of ACT in solid tumors while also ensuring its effectiveness and safety [13].

Tumor-infiltrating lymphocyte (TIL) treatment and chimeric antigen receptor (CAR) T cell therapy are the two primary forms of ACT. The first step in TIL treatment is surgical excision of the patient’s tumor. This tumor tissue contains immune cells, including T cells that had naturally invaded the tumor. These T cells are specific to the patient’s cancer and have the ability to infiltrate the tumor microenvironment. These TILs are expanded and cultivated in vitro in the laboratory. In order to increase the quantity of TILs, many growth factors are employed. The expanded TILs are often activated to enhance their anti-tumor effectiveness. Once a sufficient number of activated TILs is obtained, they are re-infused into the patient. These TILs are intended to recognize and target cancer cells throughout the body. The reinfused TILs not only penetrate into the tumor areas but also circulate throughout the patient’s bloodstream. When they encounter cancer cells, TILs identify specific antigens on the surface of cancer cells and trigger an immune response that destroys them [13].

TIL therapy has demonstrated encouraging outcomes, especially when used to treat metastatic melanoma. The strategy capitalizes on the innate capacity of the patient’s immune system to identify and attach cancer cells. The goal of ongoing research is to increase the overall success of TIL therapy by optimizing and expanding its applicability to other types of cancer [23]. There are some limitations associated with traditional T-cell-based treatments. For example, TIL therapy entails the surgical excision of tumor tissue, and obtaining a sufficient number of TILs can be challenging in some cases, particularly types of solid tumors. Not all patients have T cells that have already infiltrated. TILs harvested from tumors may already be exhausted or functionally impaired due to prolonged exposure to the tumor microenvironment. This limits their effectiveness in recognizing and killing cancer cells. In addition, TILs are a heterogeneous population, and not all TILs may have specificity for cancer antigens. Ensuring that the isolated TILs can effectively target and kill cancer cells is a key challenge. Both the TIL and TCR therapies, however, can only target and eradicate cancer cells that present antigens expressed on the MHC. To overcome this limitation, engineered T cells (e.g., CAR) are required that are not restricted by the MHC [13].

In engineered T cell receptors (eTCRs), the patient’s T cells are altered to express a novel T cell receptor. The novel TCRs allow T cells to target specific cancer antigens. TCRs are restricted because they can only respond to antigens presented by their own MHC, rather than all antigens on a tumor cell’s surface. Due to MHC restriction, there are some limitations of eTCR therapy, including the following: (1) cancer cells can avoid T cell recognition by downregulating MHC molecules, rendering themselves invisible to T cells, (2) there is a lack of antigen diversity, and (3) TCR therapy is dependent on the compatibility and availability of MHC molecules, which vary between individuals [24]. Scientists can overcome these limitations by developing engineered T cells with modified immune properties and multiple receptors to achieve better outcomes for cancer patients. The development of chimeric antigens receptors (CARs) can bypass requirement of the MHC and instead target a specific antigen present on the tumor cell [25]. The major difference between engineered TCR and CAR-T cells is the type of receptor inserted into the T cells. Engineered TCRs use naturally occurring or slightly modified T cell receptors that can bind to antigens displayed by MHC molecules on tumor cells, whereas CAR-T cell therapy employs an artificial receptor that combines the antigen-binding component of an antibody with the signaling part of a TCR. T cells from the patient or allogenic donor are genetically altered in vitro to incorporate CARs before being infused into the patient. This latter strategy overcomes the related problem.

Adoptive cell therapy employs immune system cells such as dendritic cells and natural killer cells and bispecific antibodies to combat cancer. One of the potentially effective cancer immunotherapy strategies is the adoptive transfer of monocyte-derived dendritic cells (MoDCs). The process entails removing monocytes from the blood of the patient, differentiating them into MoDCs in the laboratory, and either electroporating them with RNA or loading them with tumor antigens. After being reintroduced into the patient, these antigen-loaded MoDCs can activate the immune system to combat cancer cells. The MoDCs enter the patient’s body and travel to the lymph nodes, where they engage in T cell interaction. The T cells are activated and primed to attack cancer cells when the MoDCs expose them to the tumor antigens. Treatment for cancer may be greatly enhanced by the adoptive transfer of MoDCs. Since the MoDCs are made from the patient’s own blood, the therapy is customized for each individual. Additionally, this lessens the possibility of graft-versus-host disease (GVHD), a major side effect of certain other immunotherapies [26].

NK cells, another component of the immune system, supplement the MHC-restricted tumor lysis carried out by cytotoxic T cells by performing cytotoxic tasks without showing preference for specific MHC molecules. Recent research has demonstrated that NK cells functioned poorly in the TME, exhibiting decreased cytotoxic efficacy and a changed production of proinflammatory cytokines. The adoptive transfer of autologous NK cells is the transfusion of ex vivo activated and expanded NK cells into patients. Several NK-based immunotherapies include CAR-NK cell therapies, in which engineered natural killer cells (NK cells) are transfused to express CARs against a particular tumor antigen; cytokine therapies, in which NK cell activity is increased by the infusion of particular cytokines; and mAb-based treatments, which denote the administration of antibodies to obstruct inhibitory receptors of NK cells [27]. The blockage of inhibitory receptors on NK cells shows promise in a similar manner to ICIs, which block inhibitory pathways in T cells. Several NK cell inhibitory receptors have been investigated for their therapeutic potential and clinical application [28]. The primary inhibitory receptors on human NK cells are the killer immunoglobulin receptor (KIR) family and the CD94/NKG2A heterodimer. Antibodies that target KIRs, either on their own or in conjunction with other therapeutic drugs, can increase the antitumor activity of NK cells. Furthermore, it has been observed that antibodies that specifically target NKG2A are likewise successful in inducing NK cell responses. Currently, clinical trials are assessing the anticancer efficaciousness of monalizumab, a new anti-NKG2A antibody. Importantly, activating receptors might also be used in addition to inhibitory receptors. For example, cytokines could be used to increase the expression of activating receptors or antibodies could be delivered to target cells to induce NK cytotoxicity [29].

Bispecific antibodies (BsAbs) identify two distinct antigens in a single molecule, and this has prompted research in academia and the pharmaceutical industry. The most promising BsAb among the several BsAbs is T-cell-engaging BsAb (TCEB), a novel class of therapeutic medicines intended to attach to tumor cells and T cells simultaneously via tumor-cell-specific antigens in immunotherapy. Although hematological malignancies respond well to chimeric antigen receptor (CAR)-T cell treatment, adverse effects such as CRS, neurotoxicity, and on-target off-tumor consequences have also been reported. Studies carried out to stimulate T cells ex vivo with BsAbs are underway to decrease side effects. When T cells were primed ex vivo by BsAbs, the therapy produced strong antitumor responses, effectively infiltrated the tumor site, and released fewer cytokines than when BsAb and T cells were infused separately. This minimized systemic side effects. OKT3×hu3F8 BsAb-armed T cells (GD2BATs) selectively killed GD2-positive osteosarcoma and neuroblastoma cell lines in vitro. GD2BATs demonstrated results in certain patients without appreciable side effects in phase I studies for patients with GD2-positive tumors. In recent research, the BsAb arming strategy was combined with TCR signaling CD3ζ domains to induce non-MHC-restricted cytotoxicity in headless CAR-T (hCART) cells. These cells lack extracellular scFv CAR domains and only contain the transmembrane, the intracellular domain of co-stimulatory receptors. The capacity of hCART cells to target is ascertained by equipping them with BsAbs; these may include one or more BsAbs to target various tumor antigens. Conventional IgG-based antibodies are unable to establish innovative modes of action and therapeutic uses that are made possible by bispecific antibodies (bsAbs). There has been much interest in the creation of these molecules over the previous ten years, and, by the end of 2023, 14 bsAbs had been approved, 11 for the treatment of cancer and 3 for applications outside of oncology. Different forms, targets, and molecular processes are utilized by bsAbs to mediate their anticancer effect [30].

### 3.5. Chimeric Antigen Receptor T Cell (CAR-T) Therapy

**Figure 1:**
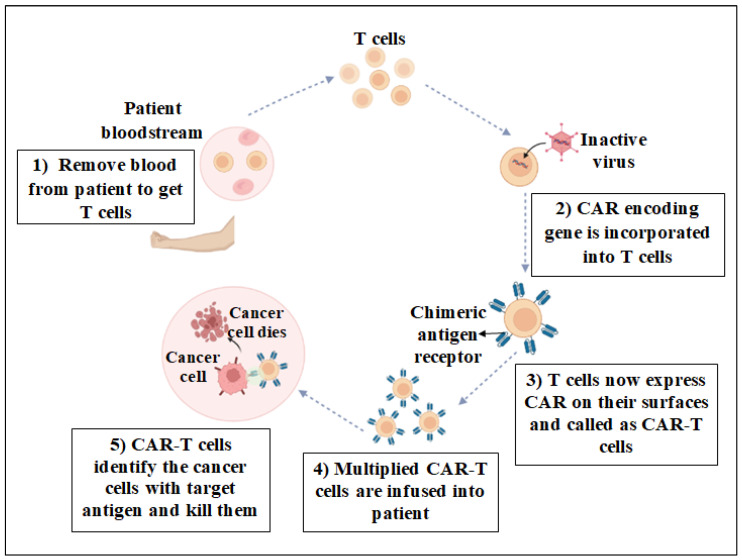
CAR-T cell therapy. T cells are taken from a patient, engineered to target cancer with a CAR gene, and then multiplied. These CAR-T cells are returned to the patient to target and combat cancer cells.

CAR-T therapy has emerged as one the most successful and groundbreaking types of ACT, specifically for the treatment of blood cancers. This innovative approach involves the therapeutic transfer of adoptive T cells into cancer patients. Adoptive T cells are engineered T cells that express artificial receptors. In CAR-T cell therapy, a patient’s natural T cells are extracted from peripheral blood and are genetically modified by adding a synthetic chimeric antigen receptor (CAR). Following this modification, the CAR-T cells are culture-expanded and amplified in vitro before being reinfused into patients as a cancer treatment. Once injected, these genetically modified CAR-T cells effectively identify and eliminate cells expressing a tumor-associated antigen (Figure 1). The efficiency of CARs as a cancer treatment is evaluated by counting the number of cancer cells in the blood and bone marrow of the patient, as well as performing imaging tests to determine tumor size. The outcome may vary from patient to patient and may take some time to manifest [6].

CAR-T therapy has revolutionized cancer treatment by addressing some of the limitations of old immunotherapies. The targets of CAR-T therapy are not only specific but also multiple antigens on cancer cells. In addition, it bypasses the requirement of MHC molecules and develops long-lasting immunity, which helps in the prevention of cancer recurrence. While older immunotherapies were effective against blood cancers, CAR-T treatment has the potential to treat solid tumors as well. CAR-T cell production can be enhanced to increase its potential in a number of solid tumors, despite its current limitations. Scientists have improved CARs and tested new approaches using tumor-specific antigens (TSAs) and tumor-associated antigens (TAAs). The precision, effectiveness, and possibility for solid tumors make CAR-T therapy an ideal treatment option for cancers.

The key features that contribute to the success of CAR-T cell therapy are the following:

1. It shows success in treating specific hematological cancers, including diffuse large B cell lymphoma (DLBCL) and B cell acute lymphoblastic leukemia (B-ALL). It has resulted in high response rates and, in some cases, long-term remissions.
2. CAR-T cells are engineered to identify and target specific antigens on the surface of cancer cells. The therapy can be targeted at cancer cells while maintaining normal, healthy cells by tailoring the CARs to a specific antigen.
3. CAR-T therapy involves the patient’s own T cells followed by their genetic modification to express CAR specific to cancer cells and their re-infusion into the patient. This tailored approach improves the specificity of the treatment and reduces some side effects.
4. Unlike traditional T cell treatments that depend on MHC presentation, CAR-T cells have the ability to detect antigens without the need of the MHC. This fact is particularly beneficial when MHC expression is downregulated by cancer cells.
5. In some patients, CAR-T therapy has exhibited durable responses, meaning persistent benefits over a long period of time, potentially providing long-term remission.
6. Certain types of CAR-T therapies (such as Kymriah (tisagenlecleucel) and Yescarta (axicabtagene ciloleucel) have received approval from regulatory agencies, including the U.S. Food and Drug Administration (FDA), for the treatment of specific types of leukemia and lymphoma, which allows for insurance reimbursement.
7. Ongoing research focuses on improving CAR design, addressing limitations, and expanding the application of CAR-T therapy to other cancer types. Next-generation CARs are being developed to enhance safety, efficacy, and applicability.

CAR-T cells are engineered to identify and target specific antigens on the surface of cancer cells, offering a tailored approach that spares normal, healthy cells. This precision is due to the CAR design, which is specific to an antigen found on cancer cells, ensuring that CAR-T cell treatment has long-lasting effects. These cells can persist in the body, providing ongoing surveillance and the potential to combat cancer cells if they reappear. Compared to other available treatments, such as chemotherapy or radiation therapy, CAR-T therapy offers numerous advantages, particularly for blood cancers. For example, CAR-T cells can detect antigens without the need for MHC presentation, a significant benefit when cancer cells downregulate MHC expression to evade the immune system. This capability is important, especially when traditional treatments fail to yield long-term remission or cure, highlighting the remarkable promise of CRA-T therapy for such challenges. As a one-time treatment, CAR-T therapy involves extracting T cells from the patient, engineering them to become CAR-T cells, and then reintroducing them into the patient’s body. This process can significantly reduce the duration and intensity of treatment compared to ongoing chemotherapy and radiation sessions. Additionally, CAR-T therapy avoids the severe side effects associated with intensive chemotherapy regimens used during stem cell transplantation, potentially improving the patient’s quality of life during treatment. Once infused, CAR-T cells act as living drugs, remaining in the body to monitor and eliminate any emerging cancer cells, thus providing a long-term defense. Currently, CAR-T cell therapy is approved for patients where a transplant is not expected to be curative or for those who have relapsed post-transplant. However, its efficacy and unique benefits suggest that it may eventually supplant many forms of transplantation [31,32]. CAR-T therapy, initially successful in cancer therapy, could now become a therapeutic option for some autoimmune diseases. Additionally, preclinical models have produced regulatory CAR-T cells and chimeric autoantibody receptor T cells to target certain autoantibodies or alter autoreactive immune cells. Clinical outcomes include B cell elimination in systemic lupus, systemic sclerosis, rheumatoid arthritis, and myasthenia gravis using anti-CD19 and anti-B-cell maturation antigen CAR-T cells [33].

#### 3.5.1. CAR-T Structure

CARs, which are engineered receptors, can activate the immune system against cancerous cells by regulating T cell receptors. These receptors primarily consist of (a) an extracellular antigen-binding domain, (b) a spacer or hinge region, (c) a transmembrane domain, and (d) one or several intracellular signaling domains. Once the synthetic domains are packaged into viral vectors, they are then integrated into T cells expressing the domains on their surface. Afterward, these modified cells are administered to cancer patients, triggering the immune system against tumor cells.

#### 3.5.2. Antigen-Binding Domain

The extracellular antigen-binding domain is a crucial component of the CAR construct. This domain imparts specificity for the target antigen as it is responsible for recognizing and binding to specific antigens present on the surface of target cancer cells. This domain is usually derived from the light (VL) and heavy (VH) chains of mAbs, which are joined by a flexible, synthetic, single-chain antibody fragment to form a single-chain variable fragment (scFv). The scFv of the CAR binds to the antigens on the extracellular surface of cancer cells, activating T cells without the need for MHC molecules. However, several features of the scFv significantly affect CAR performance beyond simply identifying and binding the target epitope [34]. The scFv of the CAR affects its function not only by identifying and binding the target epitope but also by other factors, such as the mode of association between the VL and VH chains, the positions of the complementarity-determining regions, the antigen-binding affinity, the target antigen density, the epitope location, and the avoidance of ligand-independent tonic signaling [35]. These factors influence the specificity, efficacy, and safety of the CAR-T cells. Therefore, the antigen-binding domain of the CAR must be carefully designed to optimize its interaction with its target antigen [36,37].

#### 3.5.3. Hinge Region

The spacer or hinge region is a structural region on the extracellular surface that joins the antigen-binding region to the transmembrane domain. It allows for flexibility and length for the antigen-binding domain to control steric hindrance and reach the targeted epitope. The hinge region also affects CAR signaling, expression, flexibility, and epitope recognition depending on its composition and length [38]. Moreover, the spacer length is important for creating sufficient intercellular space for the formation of the immunological synapse [39]. Long spacers can bind complex glycosylated antigens or membrane-proximal epitopes better, while short spacers can access membrane-distal epitopes more easily [40]. However, the optimal spacer length varies for each pair of antigen-binding domains and must be determined empirically. The most common hinge regions are acquired from amino acid sequences of CD8, CD28, IgG1, or IgG4. Spacers derived from IgG can cause the depletion of CAR-T cells and reduce persistence in vivo by interacting with Fcγ receptors. However, these effects can be prevented by further developing the spacer region depending on specific structural or functional requirements [41].

#### 3.5.4. Transmembrane Domain

The transmembrane domain is a hydrophobic area that attaches the CAR to the T cell membrane and is the least characterized region of all CAR components. It helps in maintaining the structural integrity of the CARs and facilitates signal transduction, the CAR expression level, synapse formation, and dimerization with endogenous signaling molecules. The transmembrane domain is commonly produced from natural proteins such as CD28, CD4, CD8α, or CD3ζ [6]. However, its effect on CAR function is not completely understood, as it is often modified depending on the requirements of the intracellular signaling domains or extracellular spacer region. For example, the CD3ζ transmembrane enhances CAR-mediated T cell activation by enabling CAR dimerization and integration into endogenous TCRs [42]. However, it also reduces CAR stability, unlike the CD28 transmembrane [43]. Similarly, transmembrane domains and CD8α hinges decrease IFNγ and TNF secretion and the activation-induced cell death (AICD) of CAR-T cells compared to CD28-derived domains. Therefore, the spacer region and transmembrane domains affect the production of cytokines and the activation-induced cell death of CAR-T cells. Studies recommend that the optimal signaling of CAR-T cells may require joining the transmembrane domain with the proximal intracellular domain, while the common CD28 or CD8α transmembrane domains can improve CAR stability and expression [43].

#### 3.5.5. Intracellular Signaling Domain(s)

The intracellular signaling domain has received the most interest in CAR design for developing effective antitumor immunity. It is an important part of the CAR construct because it determines CAR-T cell effector and activation pathways. The intracellular signaling domain of CAR-T plays a crucial role in transmitting signals upon recognition of the target antigen. This domain consists of components that activate various signaling pathways within the T cell, leading to T cell activation, proliferation, and the destruction of target cells. The primary intracellular signaling domain components are made up of co-stimulatory molecules and one or more T-cell-receptor-derived signaling molecules. The CD3ζ chain is a component of the T cell receptor (TCR) complex and is a fundamental signaling molecule that activates T cells and produces cytokines. It includes immunoreceptor tyrosine-based activation motifs (ITAMs) that are phosphorylated upon antigen detection. Phosphorylated ITAMs serve as docking sites for signaling molecules and initiate downstream signaling cascades. Many CARs also include co-stimulatory domains to provide additional signals for T cell activation and enhancement of function. Two common co-stimulatory domains employed in CAR-T therapy are CD28 and 4-1BB (CD137). The CD28 domain enhances the early activation and effector functions of CAR-T cells and stimulates T cell proliferation and cytokine production. The 4-1BB domain is associated with improved CAR-T cell persistence and memory. Signaling through 4-1BB enhances the persistence of the CAR-T cell response. These molecules can provide signals to the CAR-T cells that promote cell survival, increase cytokine secretion, induce memory function, and boost metabolic activity. The combination of diverse signaling molecules can influence CAR-T cell function and phenotype, enhancing their antitumor effects. Different signaling molecules may have different pros and cons in different tumor settings. Research on the ideal configuration and composition of the intracellular signaling domain is still going on, with the aim of developing more powerful CAR-T cells to combat cancer while minimizing its side effects.

## 4. Evolution of CAR-T Cells

**Figure 2:**
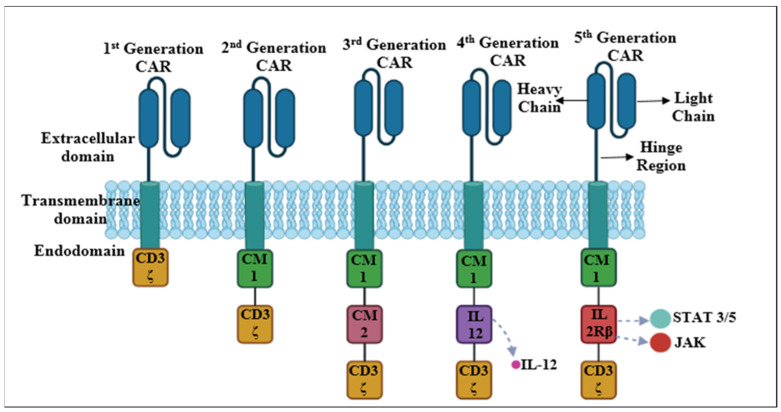
Generations of CAR-T cells. CM, co-stimulatory molecule. IL, interleukin. CAR, chimeric antigen receptor. The figure shows the evolution of CAR-T cells over five successive generations. First-generation CARs exclusively have CD3ζ-derived signaling modules. Second-generation CARs include a CD3ζ-derived signaling module and a co-stimulatory domain. Third-generation CARs have a CD3ζ-derived signaling module and two co-stimulatory domains. Fourth-generation CARs have a CD3ζ-derived signaling module, a co-stimulatory domain, and IL-12 production module. Fifth-generation CAR-T cells are made up of CD3ζ-derived signaling module, a co-stimulatory domain, and IL 2Rβ production module that is also involved in JAK-STAT pathway.

CAR-T cells have evolved through many generations based on the modification of the intracellular domain since its discovery in 1989. Figure 2 depicts different generations of CARs and their endodomain components. The main features of each generation have been discussed below.

### 4.1. First-Generation CARs

First-generation CAR-T cells were the initial versions of CARs designed as cancer immunotherapy. These molecules marked the beginning of the development of CAR-T cell technology. The first-generation CARs have a simple endodomain structure made up of either the CD3ζ-chain or FcεRIγ chain, which are the primary signal transducers of the endogenous TCR. These CARs failed to produce sufficient endogenous interleukin-2 (IL-2) for the survival and proliferation of T cells. Therefore, exogenous IL-2 was required to eradicate the tumor. The addition of exogenous cytokines enhanced the performance of first-generation CARs through various, aspects such as the following:

8. Exogenous IL-2 stimulates the survival and persistence of CAR-T cells in the tumor microenvironment by inhibiting premature cell death and improving their antitumor ability;
9. It enhances CAR-T cell growth, resulting in a bigger pool of effector cells capable of targeting tumor cells;
10. It stimulates the differentiation of regulatory T cells (Tregs) and effector T cells. It is important to balance these subgroups for efficient antitumor immunity.
11. It improves the capacity of CAR-T cells to identify and combat cancer cells.

Further studies demonstrated that removing the phosphorylation sites of ITAM A and C in the CD3ζ signaling domain could reduce the apoptotic signal that affects transgene expression. Despite this finding, most clinical trials used CAR-T cells with the CD3ζ-chain rather than the FcεRIγ-chain because the former had three ITAMs while the latter had only one [44]. Although having lower expression levels in vitro, CAR-T cells with the CD3ζ-chain were more successful at triggering T cells and eliminating tumor cells. The transmembrane domain of CAR-T cells was composed of a homologous or heterologous dimer of CD28, CD8, or CD3, which could facilitate cellular activation by dimerizing and interacting with the endogenous TCR [45].

Different tumors were treated with first-generation CAR-T cells targeting different antigens, such as the alpha-folate receptor (FR) CAR-T cells, carcinoembryonic Ag-specific CD3ζ (MFEζ) CAR-T cells, scFv(G250) CAR-T cells, CE7R CAR-T cells, CD10 CAR-T cells, and GD2 CAR-T cells. However, most of these trials did not achieve satisfactory results due to insufficient proliferation, short in vivo life expectancy, and low cytokine secretion by the first-generation CAR-T cells [46].

### 4.2. Second-Generation CARs

The second-generation CARs improved the endo-domain structure by adding a co-stimulatory domain to the CD3ζ-chain, which provided the primary signal for T cell activation. The co-stimulatory domain can be derived from different types of receptors, such as CD137 (4-1BB), CD134 (OX40), or CD28, which produce the second signal for T cell activation. The second signal is essential for producing IL-2, a cytokine that prevents apoptosis and enhances T cell function. Without the second signal, T cells cannot respond effectively to the antigen even if they recognize it through the TCR, which binds to the MHC–antigenic peptide complex on the antigen-presenting cells (APCs) [47,48]. The addition of the co-stimulatory domain to the CAR-T cells increased their cytotoxicity, proliferation, persistence, and memory in vivo compared to the first-generation CARs. The co-stimulatory domain also influenced the proliferation and persistence of effector and memory T cells. For example, CD28 promoted lymphocyte expansion and development, CD134 enhanced IL-2 production and sustained proliferation, and CD137 maintained the T cell response and memory of CD8+ T cells [49]. Several trials used second-generation CAR-T cells that targeted CD19, a marker of B cell malignancies, with different co-stimulatory domains, such as 4-1BB or CD28 [50]. Although direct comparisons are lacking, studies suggested that 4-1BBζ-CAR-T cells had a longer survival than CD28ζ-CAR-T cells. However, CD28ζ-CAR-T cells had more autonomous proliferation, stimulation, and growth while 4-1BBζ-CAR-T cells might suffer from early depletion that could impair their antitumor activity [51].

### 4.3. Third-Generation CARs

The third-generation CARs were developed to enhance efficiency. The third-generation CARs enhanced the endodomain structure by combining two co-stimulatory domains (i.e., CD28-41BB or CD28-OX40) with the CD3ζ-chain, which increased their potency, cytokine production, and cytotoxicity [52]. The primary concern was whether or not the third-generation CARs had superior characteristics than the second-generation CARs. Ramos et al. (2018) conducted an in vivo study comparing the third-generation CD19-specific CAR-T cells with second-generation CD19-specific CAR-T cells in B cell non-Hodgkin’s lymphomas. They employed two distinct constructs: one that had only the co-stimulatory domain CD28 and the other that contained both CD28 and 4-1BB. The results of the study demonstrated that third-generation CAR-T cells had a longer persistence (up to 40 times) and better expansion than second-generation cells [53]. Additionally, preliminary trial data did not indicate significant increases in response rates over traditional CAR-T treatments, despite positive overall outcomes. The currently available data are inadequate and have been derived from heterogeneous populations; therefore, conclusive decisions could not be made. However, third-generation CAR-T treatments still have a number of shortcomings compared to the first generations, such as difficulties in manufacturing or inadequate effectiveness, which justifies research into CAR-T cells from the subsequent generations [53].

In a pilot clinical trial, researchers tested third-generation CD20-specific CARs with CD28 and 4-1BB co-stimulatory domains for lymphoma patients. The trial enrolled four patients with mantle cell lymphoma. Three patients received the CAR-T cell treatment after standard chemotherapy. The treatment was well tolerated, and two patients with no detectable illness remained disease-free for 12 to 24 months, respectively. However, at 12 months, the third patient experienced a partial response and relapsed. The modified CAR-T cells were identified in the patient’s blood for up to a year, but at low concentrations. These third-generation CARs showed some anti-tumor effects but they did not show significant improvement over the second generation. One possible reason for the lack of improvement is the fewer number of studies conducted with these CAR-T cells compared to the second-generation CARs. More research is required to optimize the selection of co-stimulatory molecules, which are essential for enhancing the activation, proliferation, and survival of CAR-T cells. Moreover, the effectiveness and safety of these therapies need to be evaluated in diverse patient groups.

### 4.4. Fourth-Generation CARs

Fourth-generation CARs are also known as TRUCKs (T cells redirected for universal cytokine-mediated death). Fourth-generation CARs were built on second-generation CARs (containing 1-3 ITAMs) and associated with a constitutively or inducibly produced chemokine (e.g., IL-12). This combination allowed them to secrete cytokines that can trigger innate immune cells to eradicate antigen-negative cancer cells that escaped the CAR-T cell recognition and enhance the activation of T cells in the tumor site. The potential of TRUCKs to modulate the tumor microenvironment by inducing the expression of transgenic immune modifiers (i.e., cytokines) is worth exploring. Above all, this transgenic cytokine is released and stored in the CAR-T cells and only obtained when it is induced. Furthermore, the fourth-generation CARs can be endowed with a self-withdrawal mechanism. For example, the activation of a suicide gene (caspase-9 gene) enables the quick withdrawal of CAR-T cells when the anti-tumor impact is attained. TRUCKs have been applied to autoimmune disorders, metabolic conditions, and viral infections. The fourth generation of CAR-T cell therapy is gaining substantial attention in the field of cancer immunotherapy due to its highly promising potential [54].

### 4.5. Fifth-Generation CARs

The fifth generation of CAR-T cells introduced an additional intracellular domain that consisted of a truncated cytokine receptor fragment (e.g., IL-2R chain fragment) with a binding motif for transcription factors, such as STAT-3/5. This modification enabled the cells to produce a signal that not only sustained their function and memory formation but also reactivated and stimulated the immune system. IL-2 receptors activate the JAK/STAT pathway in an antigen-dependent manner. Although various approaches have been tried, the discovery of switch receptors is one of the field’s most intriguing developments. Recent investigations have shown that a drug-dependent OFF-switch leading to CAR depletion or an ON-switch leading to activation can be successfully integrated. Lenalidomide-gated CARs were developed using these principles. Even though the efficacy of these cells is marginally lower than that of previous CAR generations, greater control over the process led to an improved safety profile and an expanded therapeutic window. It is pertinent to mention here that the fifth generation of CARs is currently under evaluation [55].

## 5. Boolean Logic-Gated CARs

Boolean logic-gated CARs (BLG-CARs) are a new generation of CAR-T cells that go beyond older generations of CARs in order to regulate the activity of conventional CARs. In addition, BLG-CAR specificity has been increased concomitant with the limitations associated with conventional CARs. BLGs are digital devices that execute logical operations on binary inputs to generate outputs. The notion of BLGs enables medical devices, algorithms, and data to execute logical operations based on binary values (0 or 1) that indicate true or false statements. A BLG can assist in the design, processing, programming, analysis, development, and evaluation testing of different aspects of healthcare, including optimization, diagnosis, prevention, and treatment [56]. These state-of-the-art CAR technologies are designed to increase the effectiveness of therapy and lessen its side effects. BLG-CAR-related treatment employs engineering and synthetic biology to construct immune cells to identify and eradicate cancer cells based on the presence of dual antigens rather than one. The goal of this approach is to increase the safety and specificity of CAR-T cell treatment, particularly for solid tumors devoid of antigens specific to the tumor. In this novel method, intracellular proximal T cell signaling molecules are used instead of conventional CD3ζ domains. ZAP-70 is one such molecule that is essential for phosphorylating a leukocyte protein of 76 kDa containing the SH2 domain (SLP-76) and linker for the activation of T cells (LAT). These proteins serve as a scaffold for the proliferation of T cell signaling [57].

Though there are numerous variations of BLGs, AND-, OR-, NOT, and IF-Better logic gates are the most widely used types. The AND gate CAR system operates by dividing the different activation signals, ensuring that the main activation signal, CD3ζ, is present in one receptor construct and the co-stimulatory domains, like 4-1BB and CD28, in the other. The system is only activated when both antigens are expressed on a cancer cell, in which case T cells are transformed with CD3ζ CAR oriented toward one antigen and the co-stimulatory domain directed toward the other [58]. The OR gate CAR system uses a multi-antigen strategy that requires one or more targeted CAR-T cell antigens. For example, dual or bicistronic CARs are developed to introduce two CAR constructs in a single T cell, enabling them to target multiple tumor-associated antigens (TAAs). Additionally, CAR-T cells can be modified to express triCARs or quadCARs by this strategy to target three or more antigens. NOT logic CARs are designed to include an inhibitory signal as the endogenous signal of the off-target CAR construct rather than activation domains, and are also known as inhibitory CARs (iCARs). The NOT logic gate is capable of identifying antigens that are coupled to the signaling domain of an inhibitory co-receptor, such as CTLA-4 or PD-1, which are expressed on normal tissue but absent on cancerous tissue. In order to avoid auto-reactivity toward bystander tissues, iCARs are expressed in combination with CARs that precisely target the specific antigen [59]. The IF-Better logic gate is a unique CAR construct in which the presence of a high chimeric co-stimulatory receptor (CCR) target expression is required for killing the cancer cells, as low CAR target expression will not trigger this process. Higher CAR expression is made possible by the interaction of the CCR with the target antigen, which also improves co-stimulation. However, this effect is specifically limited to target cells that express both antigens. A preclinical study conducted on AML reported the outperformance of the IF-Better gate than both single and dual CAR-T cells in terms of anti-tumor effectiveness and prevention of tumor escape [59,60]. Boolean logic-gated CARs are a new generation of CARs that aims to target both hematological malignancies and solid cancers more precisely and safely. They are, however, not without limitations. These limitations include the possible loss of potency and efficacy due to tumor antigen heterogeneity, cost and complexity of developing the CARs, and the possibility of side effects due to the activation of the CAR-T cell by normal cells. Hence, more research is required before Boolean-gated CARs can be used routinely in clinical settings.

## 6. Manufacturing and Infusion of CAR-T Cells for Cancer Treatment

**Figure 3:**
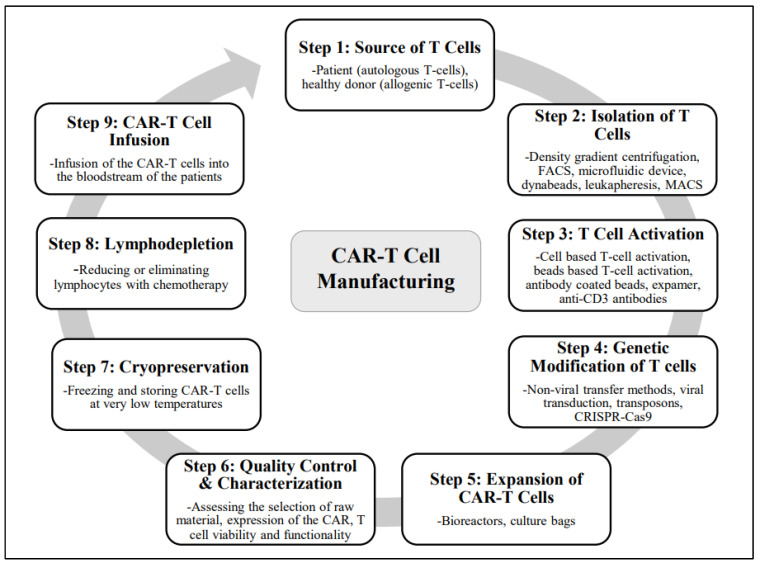
Major steps in manufacturing and infusion of CAR-T cells. CAR-T cells are isolated from an autologous or allogenic donor. CAR-T cells are activated and genetically engineered to express the CAR genes. These modified CAR-T cells are grown in bioreactors and culture bags. CAR-T cells are stored at extremely low temperatures following quality control and characterization. Chemotherapy is used to decrease or eliminate lymphocytes in selected patients, and CAR-T cells are then delivered into their bloodstream.

Since the 1970s, genetic engineering approaches have made great progress. Genetic engineering has now moved from a simple laboratory procedure to a more sophisticated approach in order to maximize transgene expression and minimize hazards. Initially, researchers employed simple laboratory methods to insert foreign DNA into cells. This included techniques such as electroporation and calcium phosphate precipitation. Although cells could be infected with genetic material (DNA, RNA) using these methods, they lacked the required specificity. With the development of CRISPR-Cas technology, genome editing has become more accurate and effective. CRISPR precisely targets DNA regions to alter genes. The manufacturing and infusion of CAR-T cells involves several key steps. Here is a brief overview of the methods involved in the manufacturing of CAR-T cells (Figure 3).

### 6.1. Sources of T Cells

The first step in the manufacturing of CAR-T cells is the collection of T cells from the patient or a healthy donor. T cells are a type of white blood cell capable of recognizing and eliminating aberrant or foreign cells. T cells isolated from the blood of the patient (autologous T cells) or from a healthy donor (allogenic T cells) can be genetically modified to express CARs using the same manufacturing process. Autologous T cells are widely used since they are specific to the immune system of each patient. This approach offers the benefit of avoiding GvHD or immunological rejection. Additionally, autologous T cells can be customized to target the specific cancer cells of the patients, which can enhance the effectiveness of therapy. Alternatively, allogenic T cells produced from the peripheral blood or umbilical cord blood of healthy donors provide a higher risk of immunological rejection or GvHD. However, it also offers the benefits of readily available T cells (off-the-shelf), thus eliminating the need for CAR production for each individual patient. Allogenic T cells can also be used for huge-scale manufacturing processes.

### 6.2. Isolation of T Cells

T cells from the blood are isolated by several methods, such as density gradient centrifugation, fluorescence-activated cell sorting (FACS), various microfluidic devices, Dynabeads, leukapheresis (this method does not isolate T cells but rather all PBMCs), and magnetic-activated cell sorting (MACS). Density gradient centrifugation involves mixing the blood with phosphate buffer saline followed by layering it on a density gradient medium and then centrifuging it. Lymphocytes (containing T cells), which are lighter, move to the interface between the density gradient media and plasma and are isolated. FACS uses fluorescence-labeled antibodies to specifically target different markers on the surface of T cells. The cells are subsequently sorted using specific equipment according to their fluorescence. Microfluidic devices selectively collect T cells while permitting other blood cells to flow through the device by using certain surface coatings and microchannels. This technique is very accurate and effective in isolating T cells. Dynabeads are magnetic beads coated with antibodies that can specifically bind to T cells. When mixed with blood, T cells expressing specific markers against the antibodies bind to antibodies, thereby adhering to the magnetic beads, and are subsequently separated by a magnetic field.

Leukapheresis is the most common method employed in the isolation of T cells from the peripheral blood of patients. During leukapheresis, blood is drawn through a needle inserted into a vein of the patient. The blood is then passed through a leukapheresis machine that isolates WBCs (including T cells) from the rest of the blood cells. The machine separates the components of blood using filtration or centrifugal force. The machine spins the blood rapidly, allowing lighter white blood cells, including T cells and plasma, to separate from heavier blood cells, such as red blood cells. Following their separation, white blood cells, including T cells, are collected and preserved for further use. The remaining blood components, such as platelets and red blood cells, are returned to the body of the patients using a second needle. After leukapheresis, T cells can be purified from other WBCs using other techniques, such as MACS and FACS. MACS relies on magnetic beads that are coated with T-cell-specific antibodies. The separation of T cells with MACS thus involves the binding of antibody-coated magnetic beads with specific markers such as CD4 or CD3 on the T cells. The labeled T cells are isolated when they pass through a magnetic field. These isolated T cells can then be used for various applications and treatment strategies, including CAR-T cell therapy or other immunotherapies [61].

### 6.3. T Cell Activation

Isolated T cells can be activated using various methods that often involve stimulation with antibodies against CD3 and CD28, as well as other activating agents. These methods include cell-based T cell activation, beads-based T cell activation, antibody-coated magnetic beads, antibody-coated nanobeads, expamer technology, and activation with anti-CD3 antibodies. This step is crucial for preparing the T cells for genetic modification. The T cells must be activated to proliferate and become susceptible to gene transfer before they can be genetically altered to express CARs. The activation of T cells requires both a primary signal from a T cell receptor and co-stimulatory signals like CD28, X40, or 4-1BB. The activation of T cells is also necessary for transducing the CAR cDNA utilizing retroviral vectors [62].

#### 6.3.1. Cell-Based T Cell Activation

This method employs APCs such as dendritic cells (DCs). APCs are intrinsic activators of T cell responses, capable of presenting antigens to T cells and stimulating the cells to activate. The APCs are produced from either the donor or patient and are loaded with anti-CD3 antibodies or tumor antigens to activate the T cells. The effectiveness of DCs differs from patient to patient as its therapeutic applications are still being researched. This constraint limits the use of DCs as a reliable source of T cell activation. Artificial antigen-presenting cells (AAPCs) are another method for activating T cells. Irradiated K562-derived AAPCs have been employed to promote the growth of CAR-T cells. However, the development and selection of AAPC lines are complicated and require significant resources.

#### 6.3.2. Beads-Based T Cell Activation

Beads-based activation employs artificial beads coated with molecules that can activate the T cells, such as anti-CD28 and anti-CD3 antibodies. The artificial beads imitate the signals that T cells receive from APCs, which can result in the strong activation and growth of T cells. Many companies have developed standard clinical-grade T cell activation reagents, including Invitrogen’s CTS Dynabeads CD3/28, Miltenyi’s MACS GMP expact Treg beads, Juno Stage Expamer Technology, and Miltenyi MACS GMP transact CD3/28 beads. These methods have significantly simplified the ex vivo activation of T cells.

#### 6.3.3. Antibody-Coated Magnetic Beads

Magnetic beads coated with anti-CD3 or anti-CD28 antibodies can activate T cells while also allowing for the easy separation of activated T cells from the beads using a magnetic field. Dynabeads CD3/28 are homogenous large paramagnetic beads that are covalently bound to CD3 and CD28 antibodies. When employed in combination with the Dynal ClinExVivo MPC magnet, this reagent has added the benefit of allowing T cells to be selected and activated in a single step. Dynabeads are the first-generation standard clinical-grade reagent for the selection and activation of CD3+ T cells. They have been widely used in preliminary clinical trials, although their availability is limited. Other commonly used antibody-coated magnetic beads for the activation of T cells include Miltenyi ExpAct Treg beads and Miltenyi MACS GMP ExpAct Treg beads. Removal of the magnetic beads is essential at the end of the production process when utilizing any beads [63].

#### 6.3.4. Antibody-Coated Nanobeads

These are nanosized beads coated with anti-CD3 and anti-CD28 antibodies, which activate T cells while simultaneously increasing gene transfer efficiency by allowing for contact between viral vectors and T cells. Miltenyi MACS GMP TransAct CD3/28 beads consist of a polymeric nanomatrix that is coupled to CD3 or CD28 monoclonal antibodies. The TransAct beads have the benefit of being biodegradable, which means that they do not need to be removed prior to use; nonetheless, upstream T cell purification is required before activation. It has been found that ExpAct Treg beads and TransAct CD3/28 beads are as effective as Dynabeads CD3/28 for CAR-T cell production [63].

#### 6.3.5. Expamer Technology

T cell activation is a key foundation in the production of T-cell-based therapies, and accurate control over T cell activation is critical in the development of next-generation T-cell-based therapies, including CAR-T cell therapies. This requirement cannot be met by currently existing approaches for T cell activation, especially not in a time-dependent manner. Expamers technology addresses these limitations. This method uses a novel platform that consists of DNA-based structures called expamers that can bind to multiple receptors on T cells and stimulate T cell activation. Expamers are diverse stimuli that are highly soluble and can be rapidly bound and released from the cell surface, thus allowing for the nearly immediate start and termination of the activation signal [64]. The Expamer, developed by Juno Therapeutics, is the most contemporary T cell activation reagent. Its distinctive core Streptamer technology has been utilized to separate viral-specific lymphocytes. Expamers allow for the precise modulation of T cell stimulation time and hold the potential of controlling T cell profiles in future products. Expamers are easily adaptable to many T cell productions and have the potential to improve the efficacy of T cell immunotherapy. The expamers can also carry the CAR gene and deliver it to the T cells using electroporation [64].

#### 6.3.6. Activation with Anti-CD3 Antibodies

This method employs soluble anti-CD3 antibodies that become attached to T cell receptors and activate them. This is a simple and extensively used method; however, it may not result in optimal T cell growth and may lead to cytokine release syndrome when used in patients. The interaction of CD3 molecules with anti-CD3 monoclonal antibodies increases T cell activation in the presence of IL-2. Studies have shown that the anti-CD3 monoclonal antibody OKT3 can effectively activate and expand the peripheral blood mononuclear cells of patient for the generation of autologous and allogenic CD19-CAR-T cells [65].

### 6.4. Genetic Modification of T Cells

Activated T cells can be genetically altered to express CARs. This modification is often performed using non-viral transfer methods, viral vectors (commonly lentiviruses or retroviruses), transposons, and CRISPR-Cas9 to introduce the CAR gene into the T cells. After the T cells are activated, they are genetically modified to express CARs, which are synthetic receptors that can identify and bind to a specific antigen on the cancer cells. The CARs consist of an extracellular domain that recognizes the antigen, a transmembrane domain that binds the CAR to the T cell membrane, and an intracellular domain that provides the activation signal to the T cell.

#### 6.4.1. Non-Viral Transfer Methods

T cells can be genetically engineered using the non-viral transfer of plasmid DNA or in vitro transcribed mRNA (IVT-mRNA), which have minimal risk of mutagenesis and low immunogenicity. Malone and his team gave the first description of mRNA for gene therapy applications in 1989, and used liposome-mediated transfection [66]. Later, in 2006, Zhao et al. reported the electroporation of mRNA to transfer TCR genes into primary T cells [67]. However, developing therapeutic approaches using mRNA faced several challenges due to its instability, negative charge, immuno-stimulatory effect, insufficient translation in the host cells, and susceptibility to degradation [68,69]. To overcome these obstacles, researchers have gained a better knowledge of the relationship between the stability and structure of mRNA and developed various chemical modification techniques. Some of the structural modifications of mRNA include anti-reverse cap analogues (ARCAs) and a polyadenylate tail, which enhance the stability and translation efficacy of mRNA. The poly (A) tail should have more than 100 residues for optimal results. Another modification is replacing the less stable 3′UTR and 5′UTR from the β-globin gene with adenylate–uridylate rice elements (AREs), which are common mRNA degradation signals found in the 3′UTRs of most eukaryotic mRNAs. When AREs are substituted for the 3′UTR of a more stable mRNA, like the mRNA for β-globin, the stability is increased. These modifications make mRNA more stable and allow for longer-lasting expression [68].

mRNA can function in the cytoplasm without entering the nucleus. It is possible to produce IVT-mRNA with structural alterations that can enhance its stability. Therefore, advanced delivery methods are required for the development of mRNA as a therapeutic tool. Protamine-mRNA, polymeric, lipid–polymer hybrid, lipid, and gold nanoparticles are the different types of nanoparticles that can facilitate the endocytosis and distribution of IVT-mRNA in the cells. Lipofectamine consists of cationic lipids that form liposomes with positively charged surfaces, and is frequently employed as a cationic carrier for IVT-mRNA to facilitate the mRNA entry into the eukaryotic cell via endocytosis. The negatively charged phosphate groups form complexes with these liposomes that fuse with the cytoplasmic membrane. The complex then builds up inside the cell, ruptures the endosome, and releases the genetic material into the cytoplasm for expression [70].

Electroporation employs electric pulses to create pores in the cell membrane, allowing the mRNA to enter the cytoplasm. It is one of the most effective methods for delivering the CAR IVT-mRNA construct into T cells, and this method did not result in a significant amount of electroporation-related apoptosis. Moreover, IVT-mRNA CAR electroporated NK-cell- and T-cell-induced tumor toxicity successfully in pre-clinical studies [67]. mRNA-mediated transfection technologies are safer than long-term integrated viral expression systems and allow for rapid modifications in CAR design. Despite their transient expression and shorter life expectancy, the IVT-mRNA transfection technique increases the safety of CAR-T therapy and has a clinical advantage. Without the need for suicide genes, the degradation of IVT-mRNA over time assures that the CAR is eliminated from the patient. As a result, it is simpler to convert IVT-mRNA-mediated transfection systems into a good manufacturing practice (GMP) system with less complicated release testing and potentially lower costs. In fact, patients only require a small number of repetitive CAR-T cell infusions (three to nine infusions) to produce a robust and long-lasting response. Initial clinical trials involved the use of mRNA to transfect T cells with CARs in blood cancers at the University of Pennsylvania. Additionally, an effort was made to cure solid tumors employing IVT-mRNA electroporated with CAR-T cells. However, electroporation also has some limitations and challenges that need to be addressed. One of them is irreversible electroporation, which occurs when the electric field permanently damages the cell membrane and leads to cell death. Another common adverse effect of in vivo electroporation utilizing surface plate electrodes is skin edema. Moreover, electroporation efficiency depends on the electrical characteristics of different cell types, such as size and conductivity. Smaller cells require stronger fields to be permeabilized while fat cells are less sensitive. Therefore, heterogeneous tissues may have different threshold levels for electroporation. Electroporation usually targets only the plasma membrane, but some applications may require nucleoporation, which involves higher voltage and shorter pulse lengths to penetrate the nucleus. It is preferable to use IVT-mRNA than CAR gene- and plasmid-encoded transposase because it avoids intentional incorporation into the host genome. The main drawbacks of these systems are the lengthy ex vivo culture time required to generate therapeutic doses of T cells that have been genetically modified and the serious cell damage that may occur after the electroporation of plasmid DNA [67].

#### 6.4.2. Viral Transduction

Viral transduction is the recommended method for equipping T cells with CARs. Adenovirus, retroviruses (γ-retrovirus and lentivirus), and adeno-associated virus are examples of viral transduction approaches. Retroviridae viral vectors are the commonly employed vectors in gene therapy. They provide major benefits, such as the ease of engineering of viral gene transfer vectors along with the ability to successfully incorporate genetic material into the host genome. Retrovirus vectors are used for gene transfer because of two unique characteristics: (i) transgenes of interest can replace the majority of the viral genome and (ii) the viral genome is permanently incorporated into the genome of the host cell. Due to these factors, basic retroviruses like the Moloney murine leukemia virus (Mo-MLV) were the primary effective advanced packaging system for gene transfer. The human immunodeficiency virus is the most commonly used lentiviral vector. The important genes env, gag, and pol are excised from the viral backbone and transduced in helper plasmids for viral production in order to create a CAR vector. The CAR-T transgene has been introduced in their place to replace these viral genes. Viral vector systems must show low genotoxicity, an inability to replicate, and low immunogenicity to meet safety standards in clinical settings [71]. The helper plasmids with env, gag, and pol genes and the CAR-T transgene vector are transfected into a packaging cell line to produce a stable virus-producing cell line for mass production. Then, retroviral particles are incubated with stimulated T cells (using CD28\OKT3 beads) to promote genomic integration, which allows the virion core to enter the cytosol and move along microtubules to the nucleus after the viral membranes fuse with host membrane. This technique enables the generation of T cells that express high levels of CARs. The efficiency of the CAR-T transgene during transduction via retrovirus vectors can reach up to 68% depending on the number of infected individuals. The control center for viral gene expression consists of long terminal repeats (LTRs), which function as transcription initiation (capping), an enhancer, promoter, polyadenylation signal, and transcription terminator. The 3′LTR and 5′LTR have identical sequences but the 3′LTR usually acts in polyadenylation and transcription termination rather than as a promoter [72]. One type of retroviral oncogenesis depends on the conversion of the 3′LTR and disruption of the 5′LTR into a promoter. The partial deletion of the U3 region of the 3′LTR and the replacement of the U3 region of the 5′LTR with the cytomegalovirus (CMV) promoter to initiate transcription enhances the safety of these vectors by significantly reducing the transcriptional activity from the viral LTR. However, engineered T cells may still pose risks of immune-mediated toxicity and insertional oncogenesis at random genomic locations as they persist and function over time. Moreover, these vectors have limited efficiency and capacity for gene delivery and they produce T cell populations with heterogeneous copy numbers and cytotoxic abilities depending on the expression levels of the cell surface. Another challenge for manufacturing is the high production costs of viral carriers, which may hinder the cost-effective and rapid clinical translation of virus-mediated CAR-T treatment, despite being adequate for phase I and II clinical trials [73].

#### 6.4.3. Transposons

Transposons are dual mobile genetic components made up of two plasmids that carry the transposase and the CAR transposon. To generate CAR-T cells, plasmid DNA containing the transposase and the CAR is electroporated into T cells as a transposon. The transposase removes and integrates the CAR sequence at a TA dinucleotide sequence in the target cell genome by acting on the surrounding terminal inverted repeats (TIRs). These dual vector systems can achieve stable incorporation of the transgene into T cells. Moreover, compared to conventional plasmids, transposon-mediated CAR-T therapy is less toxic, more efficient, faster, and cheaper when transfected into mammalian cells [74].

Monjezi’s team used minicircles to generate CD19 CAR-T cells. These minicircles use the non-viral sleeping beauty stable transposition of CAR genes derived from minimal supercoiled DNA vectors. Unlike LV, CAR transposons derived from minicircles integrate into safe genomic loci, minimizing mutagenesis and genotoxicity risk. The piggyback system is another transposon-mediated method that has higher gene transfer efficiency and does not insert near proto-oncogenes, resulting in functional CAR-T cells [75].

#### 6.4.4. CRISPR/Cas9

Gene/genome editing techniques have been popular among scientists since the early 2000s. The gene-editing methods involve the synthesis of restriction enzymes such as zinc fingers (ZFNs) and transcription activator-like effector nucleases (TALENs). ZFNs and TALENs can target specific genomic regions [76]. These enzymes, for example, can be employed to eliminate the natural TCR from allogenic T cells transfected with CAR-T treatment, thereby preventing the GvHD. This can be undertaken by reducing or eradicating the expression of histocompatibility antigens on the donor T cells. ZFNs and TALENs, however, face several challenges, thereby limiting the therapeutic potential of CAR-T therapy. These challenges include the following: ZFN and TALEN manufacturing can be more complex and expensive since custom protein synthesis and purification are required. This may hinder scalability and increase total manufacturing costs; ZFNs and TALENs have a more restricted targeting range than other genome editing techniques such as CRISPR/Cas9. This constraint may make it difficult to alter specific genes or parts of the genome, thereby restricting the therapeutic potential of CAR-T therapies; and ZFNs and TALENs may be more likely to cause off-target effects, which means that they may erroneously alter genes other than the intended target. This could have adverse effects that impair the safety and efficiency of CAR-T therapy [77].

CRISPR/Cas9 technology represents a significant advancement in genome editing. A short guide RNA (gRNA) directs the Cas9 protein of the type II CRISPR system to target any location of the genome and act as an endonuclease [76]. Chemical transduction, electroporation, liposome-mediated transfection, and viral vectors can all be used to deliver this enzyme to cells. The gRNA can be a component of the ribonucleoprotein(RNP)/Cas9 protein complex or a plasmid that is transcribed in mammalian cells by H1 or U6 promoters [78]. The desired transgene is subsequently incorporated in plasmid form via homology-directed repair (HDR), employing a donor template. Nanomaterials can also improve gene editing by binding the donor templates to the Cas9-modified cells with biotin–streptavidin, increasing transfer rates up to fivefold over conventional methods. Moreover, some cells show higher genomic cleavage rates when Cas9 and a single gRNA are co-injected as in vitro transcribed mRNA [79].

Using CRISPR technology, scientists have developed CAR-T cells with high homogeneity and adequate survival rates in mice models. More particularly, the cytotoxicity of the CAR-T cell was increased by incorporating the CAR sequence into the alpha constant TRAC endogenous T cell receptor locus. However, gene editing for CAR insertion is still not very efficient, with only 20% success rates and the risk of off-target mutations. The first CRISPR/Cas9 clinical trial resulted in the inactivation of the endogenous TCR and PD-1 in T cells in lung cancer patients. However, in this trial, T cells were not modified with a CAR or TCR. Similar studies using PD1-knockout autologous T cells are also being started for bladder, prostate, and renal cell carcinoma. The main challenge is the management of CAR integration, as well as the eradication of the integration of random viral delivery. Another concern is whether removing signals that inhibit T cells could cause uncontrolled cell growth or serious autoimmunity [80].

Despite CAR-T cell therapy showing promising outcomes, various challenges, including Cas9 protein immunogenicity, double-strand breaks, and off-target mutations, limit its use in clinical cancer immunotherapy. Therefore, large-scale research to assess the safety, accessibility, and effectiveness of CRISPR/Cas9-engineered CAR-T cells should be conducted. Next-generation treatments utilizing CRISPR/Cas9-engineered CAR-T cells will ultimately become more affordable, increase antitumor effectiveness, and reduce off-target toxicities [80].

### 6.5. Expansion of CAR-T Cells

Genetically modified T cells can be cultured to produce a sufficient quantity for therapeutic purposes. This step is essential for producing a significant number of CAR-T cells for a successful treatment. The genetically altered T cells are cultured in a carefully monitored lab setting to stimulate their growth and expansion. The expansion process may take a few days to several weeks depending on the mechanism of activation and gene transfer. The expansion process can also impact the CAR-T cell phenotype and function, including their exhaustion, persistence, and memory conversion. Therefore, they must be cultured in specific media that contain growth factors and cytokines to promote cell division. The CAR-T cells are extracted, processed, and formulated once they have reached the desired level of expansion before being infused into the patient [61,62].

CAR-T cell production can be efficiently and carefully scaled up with the use of specialized systems like culture bags and bioreactors. Compared to conventional laboratory flasks, these systems offer better control over the growth conditions and a larger culture capacity. Three different bioreactor culture systems, including G-Rex, WAVE Bioreactor, and CliniMACS Prodigy, can be used to culture CAR-T cells. One of the main drawbacks of the first two systems is that cell inoculation requires opening the flask. However, the CliniMACS Prodigy system is a single device that can successfully enrich, activate, transduce, and grow cells [81]. Culture bags are flexible containers designed to accommodate a greater volume of cells and culture media. They are frequently used to streamline the cell culture procedure in combination with bioreactors. CAR-T cells are normally expanded in culture bags within the bioreactor. The bioreactor system is connected to culture bags, making it simple to exchange the medium and monitor the parameters of the culture. The materials used to make the culture bags are compatible for cell growth and provide the cells with a sterile environment [61,62]. Researchers and manufacturers can increase the efficiency of CAR-T cell production and increase the availability of these cells for patients in need by employing culture bags and bioreactors [61,82].

### 6.6. Quality Control and Characterization

CAR-T cells must undergo extensive quality control and characterization before being infused into patients to ensure that they meet the required specifications. This includes assessing the expression of the CAR, T cell viability, and functionality. The quality control methods may differ based on the type of CAR-T cell product and the regulatory requirements. The manufacturing environment, the quality and availability of supplementary reagents and raw materials, and donor variation have a significant effect on the quality of CAR-T cell products. In addition to being able to support the manufacturing process and carry out sufficient quality control tests, the manufacturing equipment and facilities also need to be adequately maintained. A wide range of ancillary components are employed in the generation of CAR-T cells, including formulation media, cytokines, culture media, one-time use disposables, and reagents for genetic alterations. Therefore, cellular therapies require a reliable, stable, and controlled manufacturing infrastructure to be successful. The controlled manufacturing includes the selection of raw material, in-process and end-process sampling plans, batch-production-controlled records, equipment qualification, facilities, utilities, an environment monitoring plan, and monitored quality control and analytical assays [83].

### 6.7. Cryopreservation

Cryopreservation is a method of freezing and preserving cells at extremely low temperatures to maintain their viability and functionality for future use [84]. Cryopreservation can be performed either after manufacture or before shipping. The preservation of CAR-T cells is a challenging step following quality control and characterization without losing their effectiveness. It helps to ensure the consistency and availability of CAR-T cell therapies for patients. The viability of the cryopreserved CAR-T cells has been observed to be more than 50% upon thawing. CAR-T cells that have been cryopreserved can be kept and expanded again at a later date [85].

Traditionally, cryoprotective agents (CPAs) such as dimethyl sulfoxide (DMSO), ethylene glycol, or glycerol were used to preserve CAR-T cells by preventing cell damage and ice formation at low temperatures. However, these methods encountered a few drawbacks, such as the potential toxicity of cryoprotective agents, loss of cell viability, and need for long ex vivo culture periods. Nowadays, cryopreservation techniques have been improved to overcome these constraints [86]. By maintaining the immunophenotype of the input leukapheresis, this method increases the capacity of CAR-T cells to proliferate and exhibit anticancer activity while preserving a greater number of naïve and memory stem cells. Slow freezing has been replaced with a rapid cooling method known as vitrification. Vitrification occurs at the so-called “glass transition temperature,” which is between −80 and −130 °C. It is a different physical process from freezing. Samples solidify in vitrification without forming ice crystals. This technique increases the post-thaw viability and functioning of CAR-T cells while reducing cryoinjury and CPA toxicity. The most effective methods of combating the toxicity of CPAs in small tissues involve combining different CPAs to lessen the toxic effects of each agent separately, using CPAs with mutual toxicity neutralization effects, employing CPAs with weak water interactions to reduce the disruption of hydration layers surrounding biomolecules, and lowering the concentrations of penetrating CPAs by combining them with non-penetrating CPAs and ice blockers [87,88]. Nevertheless, there are still difficulties in scaling up the use of cryopreservation for complex tissues and huge volumes [89]. Consequently, in order to define the optimum procedures and guidelines for CAR-T cell cryopreservation, more investigation and development are required.

### 6.8. Lymphodepletion

Lymphodepletion is a process in which lymphocytes are either depleted or their number is reduced before receiving CAR-T therapy. This phase is also an important part of CAR-T cell therapy as it increases the survival and potency of infused CAR-T cells. Prior to CAR-T cell infusion, patients undergo a conditioning regimen, which involves high-dose chemotherapy or a combination of radiation and chemotherapy. This regimen reduces both healthy and malignant lymphocytes in the patient. When the lymphocyte count is reduced, the space frees up for the infused CAR-T cells to proliferate and exert their therapeutic effect. With fewer competing lymphocytes, CAR-T cells have a better chance to survive. The infused CAR-T cells continuously target the cancer cells for a long period of time. A possible side effect of CAR-T cell therapy called cytokine release syndrome (CRS) can also be managed with the use of lymphodepletion. Lymphodepletion reduces the severity of CRS by decreasing the quantity of lymphocytes, thus contributing to the release of cytokines. Lymphodepletion can help to lessen the severity of CRS by decreasing the quantity of lymphocytes that can contribute to the release of cytokines. Lymphodepletion can improve the engraftment, expansion, and function of the CAR-T cells and reduce the immunosuppression and competition from the own T cells of patients [90,91].

### 6.9. CAR-T Cell Infusion

The final step of CAR-T cell therapy is the infusion of the CAR-T cells into the bloodstream of the patients, where they can circulate and reach the tumor sites. The cryopreserved CAR-T cells are thawed and injected back into the patient. Once in the body, these engineered T cells identify and target those cancer cells that express the specific antigen targeted by the CAR. The infusion process is usually carried out in a hospital setting, and it may take from 30 min to a few hours depending on the type and dose of CAR-T cell therapy. The patient is monitored for any adverse reactions or side effects, such as tumor lysis syndrome or any neurotoxic infection. It might take some time for the CAR-T cells to reach their full effect, and the patient might need to undergo frequent tests to monitor the durability and response of CAR-T cells in the bone marrow or blood [89].

## 7. Clinical Applications of CAR-T Cells

### 7.1. Hematological Malignancies

CAR-T cell therapy represents a significant advancement in cancer treatment, particularly in hematological malignancies such as acute myeloid leukemia (AML), lymphoma, and Hodgkin’s lymphoma. B cell maturation antigens (BCMAs) and CD19 are frequently targeted in CAR-T cell therapy for B cell cancers. However, relapse remains a common challenge, prompting ongoing investigations into alternative targets.

### 7.2. Lymphoblastic Leukemia/Lymphoma

T cell acute lymphoblastic leukemia and T cell lymphomas are malignancies of T cells. These malignancies have a poor prognosis. The poor prognosis associated with T cell acute lymphoblastic leukemia (T-ALL) and T cell lymphomas can be attributed to several factors, such as their aggressive nature, clinical heterogeneity, limited treatment options, chemo-resistance, diagnostic challenges, genetic complexity, relapse rates, and lack of specific biomarkers, making the development of precision medicine approaches more challenging. Unlike the remarkable clinical results of anti-CD19 CAR-T cell treatment for B cell cancers, the safety and efficacy of CAR-T cell therapy for T cell cancers are still under evaluation. One of the potential targets for T-ALL treatment is CD7, which is highly expressed in 95% of T-ALL patients. In an open-label, one-arm clinical trial, two T-ALL patients were treated using allogeneic anti-CD7 CAR-T cells. One of the treated patients relapsed 48 days after the treatment, while the other patient stayed in remission for more than a year [92]. According to another clinical study, anti-CD5 CAR-T cells successfully eradicated cancerous T cells. A relapsed T cell lymphoblastic lymphoma (T-LBL) patient with involvement of the central nervous system was treated in a phase I clinical trial using anti-CD5-specific CAR-T cells [93]. Preclinical studies have demonstrated that anti-CD4 CAR-T cells are more effective against T cell malignancies [94,95]. As CD7, CD5, and CD4 are also expressed on normal T cells, targeting them not only kills leukemia but also decreases the number of normal T cells [96].

TRBC1, the T cell receptor β chain constant domain 1 expressed by tumor-associated T cells, is another target for T cell malignancies. Anti-TRBC1 CAR-T cells were found to specifically eliminate malignant T cells without targeting the healthy T cells [97]. CCR9 is another innovative target for T-ALL that is not expressed on normal T cells. A study reported that only 5% of the normal T cells and over 70% of T-ALL patients express the chemokine receptor CCR9. CCR9 is also associated with poor prognosis and multidrug resistance. Therefore, it is a potential target for CCR9-positive T-ALL. Anti-CCR9 CAR-T cells were developed to target the CCR9 receptor to eliminate malignant cells. These CAR-T cells were resistant to fratricide and exhibited strong anti-leukemic efficacy [98]. Overall, in preclinical research, anti-CCR9 CAR-T cells demonstrated strong efficacy against leukemic cells. This efficacy could include the effective targeting and killing of CCR9-positive T-ALL cells. Furthermore, CD30 is a target for some subtypes of peripheral T cell lymphoma, especially anaplastic large-cell lymphoma, where it is highly expressed. Interestingly, the expression of CD30 is low on normal T cells. Patients with newly diagnosed peripheral T cell lymphomas (PTCLs) responded significantly well to the anti-CD30 antibody–drug combination brentuximab vedotin (BV), indicating that CD30 is an acceptable target for these subtypes of lymphoma. The remarkable clinical success of BV prompted the development of anti-CD30 CAR-T cells, which, in preclinical studies, displayed increased cytotoxicity against CD30-positive lymphomas [99].

### 7.3. Acute Myeloid Leukemia

Acute myeloid leukemia is the most common type of adult leukemia. However, CAR-T cell treatment has not attained similar success in AML as in acute lymphoblastic leukemia. Several targets have been studied for CAR-T cell therapy in AML, such as LILRB4, FLT3, CLL-1, Siglec-6, CD33, CD38, CD70, and CD123 [100]. CD33 and CD123 are highly expressed on leukemic stem cells but targeting them may increase the long-term risk of myelosuppression [101,102]. CD123 is a cell surface antigen often overexpressed in certain hematological malignancies, making it a potential target for CAR-T cell therapy. The administration of anti-CD123 universal CAR-T cells resulted in a quick recovery of all three patients when the co-stimulation mediated by the universal switch was withdrawn [103]. The CAR-T cells used in the treatment were described as “universal”, suggesting that they may have been engineered to target CD123 universally across patients. The term “universal switch” implies the presence of a mechanism that provides co-stimulation to enhance the activation and function of the CAR-T cells. Another stage I clinical trial used autologous anti-CD33 CAR-T cells [103]. However, all three patients died as a result of disease progression. Anti-CD38 CAR-T cell treatment has also been demonstrated in relapsed AML after allogenic hematopoietic stem cell therapy [104]. A total of 30% of patients with AML express the transmembrane tyrosine kinase known as FMS-like tyrosine kinase 3 (FLT3). FLT3-specific CAR-T cells eradicated FLT3-positive AML cells successfully and enhanced their anti-tumor effects with the inhibitor crenolanib. However, because FLT3 is expressed on hematopoietic stem cells and healthy progenitor cells, FLT3-targeted CAR-T cells can also impact normal hematopoiesis [105].

Interestingly, hematopoietic stem cells do not express CLL-1; therefore, CLL-1-specific CAR-T cells have been demonstrated to be a successful treatment for eliminating CLL-1-positive leukemia [100]. CD70 is also a potential target for AML treatment as it is not expressed on normal myeloid cells but only on AML blasts. However, the efficacy and safety of anti-CD70-specific CAR-T cell therapy are still under evaluation [106]. Siglec-6 is absent in hematopoietic stem cells and healthy progenitor cells but expressed in about 60% of AML patients. Preclinical investigations revealed that Siglec-6 CAR-T cells successfully eliminated AML blasts in a mouse xenotransplantation model of AML. Therefore, it could be a promising target for CAR-T cell treatment in AML [107]. Moreover, nucleophosmin 1 (NPM1) mutations are found in almost 35% of patients and are thought to represent the first mutations in leukemic cells. CAR-T cells that targeted a nucleophosmin neoepitope that is expressed by HLA-A2 showed potent and highly targeted anti-leukemic activity in preclinical mouse models [108].

### 7.4. Multiple Myeloma

Multiple myeloma (MM) is a lethal cancer. However, the survival rates of MM patients have been improved by innovative treatments, such as anti-CD38 monoclonal antibodies, immunomodulatory medications, and proteasome inhibitors. BCMA, which is uniquely expressed on malignant plasma cells, is one of the highest-potential treatment targets for MM [109]. The FDA has approved two anti-BCMA-specific CAR-T cell products, ciltacabtagene autoleucel (cilta-cel) and idecabtagene vicleucel (ide-cel), for the treatment of relapsed MM. They are designed to target B cell maturation antigens (BCMAs), which are highly expressed on the surface of malignant plasma cells in multiple myeloma. Although anti-BCMA CAR-T cell treatment has been successful in the treatment of relapsed/refractory(R/R) MM, BCMA expression is reduced under clinical pressure in MM patients. Therefore, more research should be evaluated to target novel antigens [110].

Several promising target antigens, such as GPRC5D, APRIL, SLAMF7, CD229, CD138, and CD38 have been studied for CAR-T cell treatment in MM patients. CD138 is present on MM cells and supports their proliferation and survival. A small clinical study using anti-CD138 CAR-T cell treatment on five patients revealed that four of them stayed stable for at least three months after achieving a clinical response [111]. CD38 is also highly expressed on activated lymphocytes and hematopoietic cells. However, anti-CD38 CAR-T cells significantly reduced tumor growth in mouse models but also negatively affected healthy lymphocytes and hematopoietic cells. Clinically, the risk of antigen escape is decreased by combining CD38 with additional targets, such as CD138 and BCMA, to produce bispecific CAR-T cells [112].

CD229 and SLAMF7 are highly expressed on MM cells. Anti-CD229 CAR-T cells have been demonstrated to successfully eradicate MM cells in preclinical studies [113]. However, SLAMF7 is also present on normal lymphocytes, such as NK cells, B cells, and activated T cells, similar to CD38. Therefore, SLAMF7 CAR-T cells can kill both CAR-T cells and normal lymphocytes. A natural ligand called APRIL can directly bind to TACI and BCMA, which are expressed on MM cells [109]. APRIL-targeted CARs can target both TACI and BCMA on MM cells, which can reduce the risk of antigen escape and enhance anti-tumor activities by preserving its trimeric conformation [114]. The G-protein-coupled receptor, class C, group 5, member D (GPRC5D), is another promising target of MM that is expressed on almost half of CD138-positive tumor cells in the bone marrow of patients. Anti-GPRC5D CAR-T cells targeted this receptor and have shown remarkable results even in patients who have relapsed after traditional cancer treatments [115].

### 7.5. Hodgkin’s Lymphoma

CD30 is a universal antigen expressed in classical Hodgkin’s lymphoma (HL). Several clinical trials have tested the safety and effectiveness of anti-CD30 CAR-T cell therapy in Hodgkin’s lymphoma. The first clinical trial from China treated six Hodgkin’s lymphoma patients with third-generation anti-CD30-specific CAR-T cells. Three of them had long-term remission of over 24 months [116]. Another clinical trial treated 27 patients with anti-CD30-specific CAR-T cells. Within 6 weeks, 67% of the patients attained complete remission. Moreover, CD30 expression in HL patients was downregulated after CAR-T cell treatment [117]. These clinical trials are still in the early stages and further large-scale validation is required.

## 8. Solid Tumors

CAR-T cell therapies have shown remarkable outcomes in patients with hematological malignancies. This finding has encouraged researchers to explore the potential of innovative CAR-T cells in solid cancers. However, solid tumors are more challenging and require different strategies and targets. Unlike blood cancers, solid tumors can suppress the expression of antigens targeted by T cells and can lack antigen-presenting mechanisms due to genomic instability. In addition, adoptive CAR-T cell treatment has shown only minimal effectiveness in treating solid tumors due to the strong immunosuppressive microenvironment, a shortage of specific antigens, tumor histopathological features, tumor heterogeneity, and insufficient trafficking of CAR-T cells to tumor sites [107,118].

### 8.1. Prostate Cancer

The PSCA antigen is frequently present in prostate cancer, which makes it a target for CAR-T cell therapy. For instance, one phase I clinical trial of CAR-T cells showed tolerability and safety in patients with PSCA-positive metastatic cancer that is resistant to treatment and suggested a phase II clinical trial [115]. Furthermore, results were compared with another study evaluating the effect of CAR-T cell dose developed to target another antigen called a prostate-specific membrane antigen (PSMA). However, a phase I clinical trial of anti-PSMA CAR-T reported that IL-2 also has a significant role in tumor destruction [119,120]. Five patients in this clinical trial received a low dose of IL-2 after treatment with lymphodepletive chemotherapy, with the aim of a ≥20% engraftment of CAR-T cells. CAR-T cell activation was inversely correlated with IL-2 depletion. No anti-CAR reactivities and toxicities were reported. The response rate of the patients was evaluated as being directly correlated with plasma IL-2 levels, inversely correlated with the stimulation of CAR-T cells, and unrelated to dose size [121].

The applications of focal CAR-T therapy instead of conventional therapies such as focal laser ablation and high-density focused ultrasound have gained much attention. Focal therapy is an alternative to conventional treatments such as surgery or radiation that aims to preserve the normal prostate tissue and reduce side effects. However, there are significant disadvantages of focal therapy, such as the lack of long-term studies and the risk of not eliminating multifocal lesions. If administered locally, CAR-T cell therapy may overcome these obstacles by selectively targeting and destroying tumor cells. Robotic biopsy systems based on MRI-TRUS fusion can be used to guide the injection of CAR-T cells into the prostate gland. The robotic device employs a combination of magnetic resonance imaging (MRI) and transrectal ultrasound (TRUS) to generate a 3D image of the prostate gland that can be used to guide the injection of CAR-T cells into the correct location in the prostate gland. The implementation of a robotic biopsy system can help in the reduction in side effects and improvement in treatment efficacy. However, more research is required to evaluate the feasibility and usefulness of this approach [122].

### 8.2. Breast Cancer

Breast cancer is the most prevalent cancer in women worldwide, and it frequently develops resistance to current available treatments. Therefore, novel therapies that can target specific molecules and antigens associated with breast cancer cells are being researched [123]. One of these antigens is mesothelin (MSLN), which is overexpressed in many solid tumors and has been shown to be a promising target for CAR-T cell treatment in breast cancer. Other potential targets include HER2, c-Met, ROR1, MUC1, NKG2D, CD133, HGFR/cMET, TROP2, CSGP4, ICAM1, TEM8, GD2, CD44v6, FRα, CEA, EpCAM, AXL, CD70, and EGFR [124].

Several studies have investigated the efficacy, safety, and tolerability of CAR-T cells specific for these antigens in breast cancer patients. However, CAR-T cell treatment for breast cancer still faces many challenges and limitations, such as toxicity, off-target effects, tumor heterogeneity, and immune evasion. More clinical trials are needed to overcome these obstacles and to bring CAR-T cell therapy closer to clinical application for breast cancer patients [125].

### 8.3. Renal and Hepatic Cancers

Two receptor kinase targets, ROR2 and AXL, are being tested for their feasibility for use in CAR-T cell treatment for early-stage renal cell carcinoma. These targets are involved in tumor migration, cell survival, and proliferation. Their efficacy and safety are being evaluated in a two-arm phase II/I clinical trial in adult patients with refractory and relapsed stage IV metastatic renal cell carcinoma. Another possible target for CAR-T cell therapy is glypican-3 (GPC3), which is expressed in hepatocellular carcinoma (HCC). Two prospective phase I clinical trials have used GPC3-specific CAR-T cells after fludarabine- and cyclophosphamide-based lymphodepletion in adults with progressive GPC3+ HCC. The results showed an overall survival rate of 10.5% at 3 years, 42% at 1 year, and 50.3% at 6 months. The combination of checkpoint inhibitors with GPC3 CAR-T therapy for PD-L1-positive HCC is now being investigated in clinical trials [126].

CD133, a type I transmembrane glycoprotein, is a promising target for CAR-T cell therapy in advanced metastatic solid tumors. A phase I clinical trial study used CD133-specific CAR-T cells in patients with various cancers (23 of which had HCC). Three HCC patients experienced partial remission, whereas fourteen experienced persistent disease. Five months was the median progression-free survival, with a disease control rate of 65.2% after three months. Repeated CAR-T infusions were found to prolong disease stability, particularly in patients who had tumor reduction after the first administration. CD147, another type I transmembrane glycoprotein, is expressed in liver cancers and stimulates tumor invasion, metastasis, and progression by activating the secretion of matrix metalloproteinase [127]. An innovative inducible control system called Tet-On can reversibly regulate the expression of CAR gene by the presence or absence of doxycycline (Dox). Xenograft experiments showed that Dox administration could control the function of CAR-T cells both in vitro and in vivo and that Dox+ Tet-CD147 CAR-T treatment effectively inhibited the progression of cancer cells [128].

### 8.4. Ovarian Cancer

CAR-T cell therapy is a powerful treatment for ovarian cancer but its effectiveness is still under investigation. One of the challenges is to target the right antigen on the cancer cells. A phase I experiment evaluated the safety of CAR-T cells that target the alpha-folate receptor (FR), which is expressed by the majority of ovarian malignancies. The trial enrolled patients with metastatic ovarian cancer who had failed platinum-based chemotherapy and infused them with FR CAR-T cells through a peripheral vein. However, the trial did not show any tumor reduction or targeted recruitment of CAR-T cells to the tumor location. Instead, it caused IL2-activation-related toxicities and a rapid decline in CAR-T cells from the blood [129]. To improve the localization of CAR-T cells to the tumor, another trial explored the direct peritoneal infusion of FR CAR-T cells, and its feasibility and safety is being evaluated in another clinical trial. The Mucin 16 (MUC16) antigen has been reported in 70% of ovarian cancer and a clinical trial is also evaluating the efficacy and safety of CAR-T cells targeting MUC16 after standard chemotherapy [130].

### 8.5. Colorectal Cancer

One of the most common tumor markers in colorectal cancer (CRC) and other solid tumors is the carcinoembryonic antigen (CEA). Targeting the CEA with CAR-T cell therapy is a potential strategy for treating relapsed or refractory CEA+ cancers. A phase I clinical trial evaluated the efficacy and safety of this approach in various CEA+ cancers including CRC patients. In this trial, CAR-T therapy was applied to 10 CRC patients. Seven out of ten patients who did not have any effect of earlier treatments had persistent disease after CAR-T cell therapy. Most patients showed a decline in serum CEA level even after long-term observation. The findings of the clinical trial showed that CEA+ CRC patients tolerated the CEA CAR-T cell treatment even in high doses and that the majority of them showed some degree of therapeutic benefit [131,132,133]. Another phase I clinical trial evaluated the tolerability and safety of CYAD-101, a CAR-T receptor encoding the natural killer group 2D (NKG2D) receptor within its intracellular domain. CYAD-101 is an allogenic CAR-T cell product that combines a peptide-based approach with the wide range of tumor targeting of the NKG2D-based chimeric antigen receptor. NKG2D binds to ligands that are frequently overexpressed in most cancers, including colorectal cancer. In this trial, fifteen patients with incurable metastatic CRC were given three doses of CYAD 101 cells after standard chemotherapy. Two patients achieved a 50% response while nine had persistent disease [134].

### 8.6. Pancreatic Cancer

Pancreatic cancer is a lethal and aggressive disease that has a limited response to existing immunotherapies such as PD-L1, PD-1, and CTLA-4 inhibitors. CAR-T cell treatment is a promising alternative to supplement previous treatment of this disease. Pancreatic ductal adenocarcinoma contributes to more than 90% of cases of pancreatic cancer [135]. PDAC has been extensively studied in clinical trials because of its high mortality rate and poor prognosis. Several antigens, such as PSCA, MUC1, claudin, CEA, and carcinoembryonic antigen-related cell adhesion molecule (CEACAM7), have been discovered as potential targets for CAR-T cell treatment in pancreatic cancer [136]. CEACAM7-specific CAR-T cells have induced remission of PDAC xenograft tumors derived from late-stage patients both in both in vivo and in vitro studies [137]. Another ongoing phase I clinical trial is testing the efficacy, safety, feasibility, and clinical activity of CAR-T cells specific for prostate stem cell antigens (PSCAs), which are expressed by some metastatic pancreatic cancers [138].

### 8.7. Thoracic Cancer

Clinical trials of CAR-T cell therapy in thoracic cancers have primarily targeted malignant pleural mesothelioma (MPM) and advanced-stage non-small cell lung cancer (NSCLC) so far. These trials are in phase I and evaluating the safety and efficacy of these targets in solid tumors, particularly lung cancer. Moreover, several of the targets previously discussed with other solid tumors, such as HER2, EGFR, ROR1, MSLN, PD-L1, MUC1, and CEA, are also currently being studied for CAR-T cell treatment for lung cancer. MSLN- and EGFR-specific CAR-T cells are higher-potential targets due to the higher specificity of the antigen and lower on-target and off-tumor toxicity [139]. Although studies have highlighted the key developments of CAR-T cells in treating solid tumors, more research would be beneficial in making evidence against this approach and treatment. Nevertheless, a more comprehensive evaluation is required to determine their long-term validity [140].

### 8.8. Solid Pediatric Tumors

CAR-T cell treatments for hematological malignancies and solid tumors represent significant advances in cancer research. Therefore, developing CAR-T cells to target pediatric solid tumors is the next frontier. Central nerve tumors are the most common solid tumors in children. Traditional chemotherapy, surgery, and radiation therapy are ineffective due to long-term negative effects. CAR-T cells have the potential to revolutionize the treatment of CNS malignancies in children. However, the drawbacks of this therapy include the heterogeneity of the tumor, the co-expression of antigens in the tumor and normal tissues, and the absence of target antigens. Investigating BTIC-specific antigens, using suitable preclinical controls, and creating novel strategies for multivalent CARs are some potential solutions. With continuing innovation and creative implementation of novel therapies, CAR-T cell therapy has the potential to improve treatment and reduce toxicity in children with CNS malignancies [141].

## 9. Registered Clinical Trials Using CAR-T Cell Therapy

CAR-T cell therapies are among the most advanced cancer treatments available today. Their development and success have led to FDA approval for treating blood cancers and expanding their potential for solid tumors. Many targeted therapies are becoming standard treatment for cancer. The success rates can vary depending on various factors, including the cancer type and individual response to therapy. While relapses occur in certain patients, the treatment shows remarkable promise for long-term survival in others. The FDA-approved anti-CD19 CAR-T cell treatments, tisagenelcleucel for the treatment of acute lymphoblastic leukemia (ALL) and axicabtagene ciloleucel for patients with lymphomas, are prime examples of the efficacy of CAR-T cell therapy in cancer treatment. In pediatric and young adult ALL, for example, CAR-T targeting CD19 and CD22 antigens has shown remarkable outcomes. Studies indicate that overall remission rates are between 60% and 32%, respectively, and that responses are sustained after a year [142]. Furthermore, in 2021, CAR-T therapy obtains another significant achievement with the FDA-approved axi-cel for the treatment of patients with non-Hodgkin’s lymphoma because of its outstanding efficacy and low rate of high-grade toxicity. When axi-cel was used to treat non-Hodgkin’s lymphoma, this phase 2 trial obtained a 94% overall response rate and an 80% complete response rate [143]. Recently approved by the FDA, BCMA-targeted CAR-T cells have an overall response rate of 81%, with 63% of patients exhibiting a full response in multiple myeloma patients [144]. A further research utilizing tisa-cel for follicular lymphoma revealed an overall survival rate of 86.2% [145]. It is pertinent to note that while CAR-T therapy can be highly effective, research is still ongoing to optimize its applications and manage its risks.

Since the approval of the first CAR-T therapy in 2017, there have been a total of 1412 registered clinical trials with different statuses, including not yet recruiting (*n* = 129), recruiting (*n* = 611), active, not recruiting (*n* = 143), completed (*n* = 102), terminated (*n* = 53), enrolling by invitation (*n* = 18), suspended (*n* = 15), withdrawn (*n* = 36), and unknown (*n* = 295) studies (www.clinicaltrials.gov). Table 1 shows the major completed trials with results (*n* = 24). The information of these clinical trials was collected from the www.clinicaltrials.gov website, dated 29 January 2024. The following search was performed: “CAR-T cell therapy for cancer” OR CAR-T cell treatment for cancer” OR “Chimeric antigen receptor therapy for cancer”.

The FDA accepted two therapies using CAR-T cells: axicabtagene ciloleucel and tisagenlecleucel, which targeted CD19 in 2017 in order to treat Hodgkin’s B cell lymphoma (HGBCL) and relapsed acute lymphoblastic leukemia (ALL), respectively. Axicabtagene ciloleucel (axi-cel) was the first FDA-approved phase II clinical trial of CAR-T therapy. The trial included 111 patients with transformed follicular lymphoma and Hodgkin’s B cell lymphoma. Patients received a single dose of axi-cel after receiving standard chemotherapy with cyclophosphamide and fludarabine. The overall response rate (ORR) after infusion was 82% and overall survival rate was 52% after a median follow-up of 18 months [146]. Tisagenlecleucel (tisa-cel) was initially reported in a phase II clinical trial for pediatric patients with ALL. The overall response rate was 81% after 3 months, 90% after 6 months, and 70% after 6 months of follow-up, respectively [147]. The second approval of tisa-cel was reported in late 2017 after a study conducted on 93 patients with HGBCL. The overall RR was 52% across all of the disease group [148]. Brexucabtagene autoleucel, the first anti-CD19 CAR-T cell therapy, was permitted for adult patients with relapsed mantle cell lymphoma (MCL) in 2020. The trial included 60 patients who had received this therapy after standard anthracycline-based chemotherapy. The ORR after infusion was 93% and overall survival rate was 83% after a median follow-up of 18 months [149].

Lisocabtagene maraleucel was accepted for diffuse large B cell lymphoma (DLBCL) in 2021. This single-arm multicenter trial included 192 patients who had received at least two or more infusions of therapy. The overall RR was 73%, with 66% patients in remission for 6–9 months [150]. All these therapies, however, are also associated with CAR-T-related toxicities such as neurotoxicity syndrome and cytokine release syndrome. Other completed and online clinical trials have also focused on targeting CD4, CD5, CD7, CD20, CD21, CD33, and CD123 for different types of blood cancer. Even if only some of these clinical trials showed encouraging results in cancer patients, many studies are evaluating other CAR-T cells targeting various antigens. Many clinical trials are also evaluating the safety and efficacy of CAR-T therapy in solid tumors such as renal cancer, pancreatic cancer, hepatocellular carcinoma, breast cancer, colorectal cancer, ovarian cancer, brain cancer, and lung cancer by targeting various antigens, including CEA, GD2, mesothelin, TnMUC1, PSCA, HER2, glypican 3(GPC3), GUCY2C, and CD19 [151,152].

IMPT-314, a Boolean logic-gated CAR-T cell therapy, is the most advanced type of CAR-T cell treatment for aggressive B cell lymphoma, including NHL as described above in the CAR generation section. The first FDA-approved clinical trial of IMPT-314 (NCT05826535) was recently launched on 9 May 2023 and is expected to be completed on 1 December 2029. There are currently 100 people enrolled in this interventional study, and more are being recruited. The study will evaluate the safety and preliminary success of IMPT-314, a drug that targets two antigens: CD20 and CD19. Although CD20 and CD19 are common targets for CAR-T cell therapy, they can also be expressed by normal B cells, resulting in undesirable side effects. By combining these two targets, IMPT-314 aims to generate a more selective and effective anti-tumor response [8]. Advancements in technologies have opened up new avenues for the development of CAR-T treatments that target novel antigens in a variety of cancers. Future generations of CAR-T treatments may be able to save patients who have failed present CAR-T therapy. Multi-antigen-targeted CAR-T therapy has the ability to address the problem of antigen escape relapses following single-antigen-targeted CAR-T treatment.

## 10. Drawbacks of CAR-T Cell Therapy

While CAR-T therapy has presented tremendous success, it is essential to acknowledge that challenges and limitations exist, including potential toxicities, a lack of efficacy in certain cancers, and the need for further optimization. Researchers are actively working on addressing these challenges and enhancing the success of CAR-T treatment to a broader range of malignancies.

### 10.1. Antigen Escape

One of the key challenges of CAR-T cell therapy is tumor resistance to single-antigen-directing CAR designs. Many patients who initially responded well to these CAR-T cells later developed malignant cells that lose or reduce the expression of the target antigen. This condition is called antigen escape. For example, CAR-T cells that target CD19 can produce lasting responses in 70–90% of refractory ALL patients, but 30–70% of them may relapse with CD19-negative disease. Similarly, BCMA expression may be downregulated in patients who receive BCMA-targeted CAR-T cells [153]. Solid tumors have also shown evidence of antigen escape. For instance, in a case study, CAR-T cell therapy for glioblastoma patients revealed that IL13Ra2 expression was decreased following tumor recurrences. To overcome this issue, many strategies have been established that target multiple antigens in CAR-T cell treatment for both blood cancers and solid tumors [154].

Simultaneously targeting several tumor antigens can be achieved by using tandem or dual CARs. Tandem CARs have two scFvs in a single CAR construct, while dual CARs have two separate CAR constructs. Both strategies can enhance the likelihood of durable remission. Some clinical trials using dual-targeted CARs (CD19/BCMA or CD19/CD22) have reported outcomes. For instance, CD19/CD22 CAR-T cell treatment has shown favorable results in adult patients with diffuse large B cell lymphoma and ALL in terms of safety and efficacy [155]. Likewise, CD19/BCMA-targeted CAR-T cells have exhibited high efficacy and favorable safety in treating multiple myeloma. Several tandem CARs, such as MUC1 and HER2 in breast cancer and IL13Ra2 in glioblastoma, have been evaluated in preclinical models in solid tumors. Dual targeting has boosted the anti-tumor responses, unlike single-antigen targeting. In the glioblastoma study, CARs targeting IL13Ra2 and HER2 decreased antigen escape and boosted anti-tumor activity. This study indicates the significance of selecting target antigens that can reduce antigen escape and enhance antitumor response to prevent relapse [156].

### 10.2. On-Target Off-Tumor Effects

On-target off-tumor toxicity ensues when genetically targeted T cells attack normal tissues that express the identical antigen as the cancerous tissue. Solid tumor antigens are often not exclusive to tumor cells but are also present on normal cells at different levels. Therefore, antigen selection is important in CAR design to balance therapeutic effectiveness and toxicity. One possible strategy to overcome this challenge is to target tumor-specific post-translational variations. Some of the targets that have been tested for CAR-T cell therapy include TAG7228, MUC1, B7-H3, and MUC16 [157]. A new type of second-generation TAG72-specific CAR-T cells is currently under evaluation after the first generation failed to produce any anti-tumor response in colorectal cancer. More research is required to develop new strategies to select antigens that can provide adequate antitumor efficacy and reduce antigen escape while minimizing toxicity issues. This approach will help to improve the clinical use of CAR-T cell therapies in solid tumors and hematological malignancies [158].

### 10.3. Tumor Infiltration and CAR-T Cell Trafficking

CAR-T cell trafficking to the target site is a vital step in mounting an effective immune response against cancer [159]. T cell trafficking is strongly associated with tumor infiltration. CAR-T cell therapy applications in solid tumors are more limited than in hematological malignancies because CAR-T cells can only travel to infiltrate solid tumors by passing through physical tumor barriers like an immunosuppressive tumor microenvironment and the tumor stroma. One way to overcome these obstacles is to use local administration instead of systemic delivery because this can (i) reduce on-target off-tumor toxicities by directing CAR-T cell activation to tumor cells and avoiding interaction with healthy tissues and (ii) eliminate the need for CAR-T cell trafficking to disease sites [160]. The intraventricular injection of CAR-T cells directed against IL13Ra2 and HER2 has demonstrated efficacy in the treatment of glioblastoma and breast cancer brain metastases in preclinical models. Likewise, a phase I clinical trial is currently being carried out based on preclinical models that showed enhanced intrapleural CAR-T cell treatment of malignant pleural mesothelioma. However, this method is only suitable for oligometastatic disease or tumor lesions, and local injection may have better efficacy [161].

The development of chemokine receptors on CAR-T cells that are compatible with and responsive to tumor-derived chemokines is another emerging technique for improving CAR-T cell trafficking. For instance, studies have demonstrated that CAR-T cells that are specific to integrin v6 and have been engineered to overexpress CXCR1 or CXCR2 can boost trafficking and significantly enhance anti-tumor activity. Physical barriers like the tumor stroma additionally prevent tumor penetration and limit the use of CAR-T cell treatment. The extracellular matrix makes up the stroma and contains heparin sulfate proteoglycan (HSPG), which CAR-T cells must knock down in order to infiltrate the tumor. Heparinase, an enzyme that breaks down heparin sulfate proteoglycan, has been added to CAR-T cells, increasing their anticancer activity and tumor invasion. Similarly, in animal models, CAR-T cells that specifically target the fibroblast activation protein (FAP) exhibit improved cytotoxic efficacy by reducing tumor fibroblasts. However, more research is needed to develop new delivery techniques to extend therapeutic efficacy to complicated solid tumors [162].

### 10.4. Immunosuppressive Microenvironment

Immunosuppressive cells are present in the tumor microenvironment and can suppress the immune system. These cells include myeloid-derived suppressor cells (MDSCs), regulatory T cells (Tregs) and tumor-associated macrophages (TAMs). Solid tumors are infiltrated by many types of cells that suppress the immune system. These cells produce growth factors, chemokines, and cytokines that facilitate tumor growth [163]. Moreover, immune checkpoint mechanisms such as CTLA-4 and PD-1 can suppress antitumor immunity. Short-term persistence can result in a poor expansion of T cells and poor or no response to CAR-T cell treatment. Co-inhibitory pathways can induce T cell exhaustion. Therefore, combining immunotherapy with checkpoint blockade and CAR-T cells is suggested to be a promising immunotherapy strategy because it provides the two components necessary to boost the immune response: (i) PD-L1/PD-1 blockade, which can maintain function, and (ii) the persistence and infiltration of T cells and CAR-T cells. In children with pre-treatment B-ALL, CD19 and PD-1 CAR-T cell therapy blocking increased CAR-T cell survival. One interesting study found that pre-treating 11 mesothelioma patients with cyclophosphamide followed by a single dosage of mesothelin and three doses of CAR-T cells targeted against the PD-1 receptor showed a 72% response rate. However, other immunotherapies may still be needed to counteract the inhibitory signals [164].

Additionally, scientists are aiming to create CARs that can withstand immunosuppressive elements, including TGF-mediated inhibitory signals in the tumor microenvironment. A different cutting-edge approach entails creating CAR-T cells capable of transmitting immunostimulatory signals in the form of cytokines, which increase proliferation, survival, and the activity of antitumor T cells. Studies that emphasize creating pro-inflammatory cytokines instead of inhibitory signals have used IL-15 expression, IL-12 secretion, and rerouting the signaling of immunosuppressive cytokines (like IL-4) toward proinflammatory cytokines. However, the combination of CAR-T cell therapy and checkpoint blockade is not enough to stimulate effector function and T cell infiltration. More research is required to evaluate the effectiveness of combining the checkpoint blockade and CAR-T cell therapy with other immunotherapies in solid tumors or complex hematological malignancies [165].

### 10.5. Toxicities Associated with CAR-T Cells

CAR-T cell therapy has presented promising results in some tumors, but it is also associated with substantial side effects that limit its usage as a first-line treatment. The type of cancer, the target antigen, and the CAR design influence the risk and severity of two major toxicities: hemophagocytic lymphohistiocytosis (HLH) and cytokine release syndrome (CRS). These toxicities have been observed in patients who were administered with the first FDA-approved CD-19-directed CAR-T cell treatment [166]. In clinical trials, some patients had potential responses to the therapy but also experienced life-threatening complications. These patients who had ALL/LBL had a massive expansion of T cells in their bodies and a very high production of cytokines. Almost all patients had some symptoms of toxicity but 23–46% of them had severe reactions. The excessive activation of immune cells and the release of toxic levels of cytokines caused (i) immune effector cell-associated neurotoxicity syndrome characterized by increased cerebrospinal fluid (CSF) and impairment to the blood–brain barrier, (ii) a hyperinflammatory syndrome, macrophage activation syndrome (MAS), characterized by high serum ferritin, CRS, liver enzymes, renal failure, hemophagocytosis, low NK cell activity and pulmonary edema, and (iii) cytokine release syndrome (CRS), characterized by a large proliferation of T cells and cytokines [167].

Patients with mild cytokine release syndrome may experience fever, diarrhea, fatigue, arthralgia, headache, myalgia, rashes, cardiac dysfunction, multiorgan system failure, hypotension, respiratory failure, and renal failure as clinical symptoms, and eventually progression to death in more severe cases [168]. Pathophysiologically, IL-6 mediates the cytokine release syndrome and thus management depends on the use of the anti-IL-6 receptor with corticosteroids and tocilizumab. However, in order to cure MAS/HLH secondary to CAR-T cell therapy, chemotherapy may be required if IL-6 suppression is ineffective. The exact incidence of MAS/HLH caused by CAR-T cell therapy is unclear, but it has been observed in 1% of patients who had the treatment [169]. The underlying mechanisms and pathophysiology of neurotoxicity are not well understood. However, its symptoms range from attention deficits, headache, confusion, focal neurological deficits, encephalopathy, or word-finding difficulties to transient coma, seizures, or life-threatening cerebral edema. Neurotoxicity secondary to CAR-T cell treatment has been reported in up to 67% of patients undergoing treatment for lymphoma and leukemia. Since IL-6 inhibitors are unsuccessful in treating CAR-T-cell-therapy-related neurotoxicity, corticosteroids are required for the management of this condition [170].

Tumor lysis syndrome (TLS) is another negative effect of CAR-T cell therapy. TLS is a group of metabolic complications that can occur when cancer cells are rapidly killed due to chemotherapy or other treatments. TLS can develop between hours to days of CAR-T cell infusion and is characterized as either clinical TLS (symptoms or organ damage caused by TLS) or laboratory TLS (abnormal blood tests without symptoms). TLS causes large amounts of cellular components, such as potassium, calcium, phosphate, and uric acid, to be released into the bloodstream. These substances have the potential to overwhelm the kidneys, resulting in acute renal damage, convulsions, arrhythmias, electrolyte imbalances, and even death. CAR-T cells cause TLS particularly in patients with a quick tumor response and high tumor burden. The risk of TLS after CAR-T cell therapy is determined by several factors, such as the stage and type of cancer, timing and dose of CAR-T cell infusion, use of prophylactic measures, and presence of other comorbidities. The prevention and management of TLS after CAR-T cell therapy involves patient selection, careful monitoring, and intervention [170]. CAR-T cell therapy is a promising treatment option for individuals with relapsed or refractory malignancies, but it must be carefully monitored and managed to avoid side effects. There are currently no approved treatments for eliminating the toxicities; hence, it is critical to improve the CAR design and investigate new approaches to eliminating CAR-induced toxicities.

### 10.6. Cost of CAR-T Therapy

One factor contributing to the high cost of CAR-T cell treatment is the expenses associated with its production and administration. With startling price tags of over USD 373,000 for a single injection, CAR-T cell therapies have produced extraordinary response rates in patients with B cell ALL since the FDA approved the first of them in 2017. The cost does not cover the costs associated with producing the medicine, handling any possible long-term side effects, or continuing other therapeutic lines following a relapse. Leukapheresis is the first stage in the multistep process of making CAR-T cells. Next, T cells are obtained through genetic engineering, which involves the use of viral vectors or non-viral ways to add CAR expression. Finally, the transformed T cells are expanded in a controlled environment [171]. Each of these procedures may call for specialized tools, knowledgeable staff, and stringent quality assurance protocols. Local cell manufacturing facilities that generate experimental goods face higher production costs due to the continued high cost of reagents and lentiviral vectors, which are often related to individual items or groups of products. This is in contrast to commercial manufacturers, who manufacture cellular therapies at scale. The customized nature of autologous CAR-T cell treatment drives up production costs. Additional logistical costs for the apheresis procedure, cryopreservation, transporting patient-derived cells to specialized manufacturing facilities, and returning the finished therapeutic product to the clinical site are also imposed by patient-specific features. This also raises the expenses associated with research and development since, though it is difficult to say by how much, the costs of conducting clinical trials for cellular therapy are probably higher than those of small-molecule medicines [172]. Numerous CAR-T cell products have been developed thus far along similar lines. Medications with encouraging early-phase data that were introduced by universities and/or small biotech businesses have frequently been acquired by large pharmaceutical companies, who have subsequently carried out the late-phase clinical trials and mass-marketed the medications [173]. There is a need for future research and advancements in CAR-T therapy to address these economic hurdles. This will pave the way not only to improving the cost-effectiveness of therapy but also to ensuring financial support for the patients and healthcare system.

## 11. Methods to Reduce CAR-T-Cell-Therapy-Related Side Effects

To achieve effective therapeutic responses, it is a requisite that the target epitope is bound by the CAR-T cell antigen-binding domain and reaches the minimal threshold level necessary to cause cytokine production and CAR-T cell activation. CAR-T cells must persist within their therapeutic window to maintain their clinical effectivity [174]. From an engineering perspective, both the degree and kinetics of CAR-T cell activation are highly affected by numerous factors, including tumor burden, the extent to which the tumor antigen is expressed on the malignant cells, co-stimulatory elements of the CAR, and the affinity of the antigen-binding domain for target epitopes. Hence, to limit toxicity and achieve optimized therapeutic levels, the CAR’s modular structure needs to be further evaluated [175].

### 11.1. Modifying CAR Structure

One method to lessen toxicity is via fluctuating the affinity of the antigen-binding domain of CAR-T cells. In cases of decreased affinity, there would be an increased requirement on tumor cells for a higher antigen density in order to achieve elevated activation levels. Consequently, this could also help to avoid targeting healthy tissues that have fewer antigens. According to some research, antigen-binding domains with micro-molar affinity were more selective for cancers with high antigen expression than those with nano-molar or sub-nano-molar affinity. Another way to control the cytokine levels produced by activated CAR-T cells is to change the transmembrane and hinge regions. For example, in a CD19-targeted CAR, changing the amino acid sequences of the transmembrane and CD8-α hinge reduced the proliferation of CAR-T cells and release of cytokines. This approach could be a useful strategy for lowering toxicity. In fact, 54.5% of B lymphoma patients receiving these modified CARs experienced a full remission in a phase I clinical trial [176].

The co-stimulatory domain is another part of the CAR design that can be adjusted depending on the tumor characteristics, toxicity concerns, and antigen density. Specifically, 4-1BB domains are linked to better T cell survival, lower toxicity, and a slower peak of T cell expansion while CD28 co-stimulatory domains are related to faster and more intense CAR-T cell activity. Consequently, 4-1BB co-stimulatory domains with lower toxicity could be more suitable for tumors with a high antigen density or high disease burden. On the other hand, CD28 co-stimulatory domains could achieve a necessary threshold level of T cell activation for the low-affinity antigen-binding domain of the CAR or low density of total surface antigens [177].

### 11.2. CAR Immunogenicity

The term “CAR immunogenicity” refers to the potential for the chimeric antigen receptor (CAR) construct to elicit immunological responses. When the host immune system recognizes the CAR constructs, it might lead to the generation of cytokine-related toxicities. However, CARs constructed using human or humanized antibody fragments rather than CARs generated from mice reduce CAR immunogenicity and the production of cytokine-related toxicities. Moreover, the transmembrane or hinge domains can also be modified for lessening the CAR’s immunogenicity and improving the survival of CAR-T cells [178].

### 11.3. Neurotoxicity and CAR-Transduced T Cell Modification

A recent possibility for reducing or preventing CAR-T cell cytokine toxicities is to change the CAR-transduced T cells themselves. Myeloid cells and cytokines are also involved in the neurotoxicity triggered by CAR-T cells as studies have found higher levels of CD14+ cells in patients with severe or grade-3 neurotoxicity. A key clinical trial on CAR-T cell therapy for large B cell lymphoma showed that CD14+ cells were more abundant in patients with grade-3 or severe neurotoxicity. Furthermore, a key clinical trial on CAR-T cell treatment for large B cell lymphoma discovered that GM-CSF elevation was the blood biomarker most closely related to neurotoxicity among those associated with severe or grade-3 neurotoxicity [179].

Experiments in animals have shown that blocking the cytokine GM-CSF, which activates monocytes and macrophages, with lenzilumab reduced the levels of CRS and neurotoxicity and improved the function of CAR-T cells. Similarly, mutating GM-CSF in CAR-transduced T cells had the same effects. These results recommend that blocking GM-CSF can help to lower neurotoxicity and CRS. Moreover, deleting or inhibiting tyrosine hydroxylase, an enzyme in myeloid cells, lowered the levels of cytokines and catecholamines. Experiments in animals also showed that blocking the IL-1 receptor reduced neuro-inflammation in mice with lymphoma/leukemia treated with CD19-targeted CARs [180].

### 11.4. CAR Off-Switches

Implementation of the suicide gene or off-switches is another promising approach to improve CAR-T cell toxicity. These switches can selectively kill engineered cells when severe events happen with the help of a secondary agent. For example, some CAR constructs can express CD20 mimotopes that make them sensitive to rituximab treatment. However, immediate reversal is necessary for this therapy to be beneficial in patients with acute cytokine-mediated toxicities. This need resulted in the development of faster switches, such as inducible Cas9, which eliminated >90% of changed T cells within 30 min in a clinical trial [181].

Other strategies have used protease-based CAR off-switches (SMASh-CARs), which can be turned off by small molecules. The main issue with CAR off-switches is that they end the therapy suddenly and may let the disease grow quickly. However, a promising strategy is to use a tyrosine kinase inhibitor such as dasatinib, which can stop T cell activation by blocking TCR signaling kinases. In animal models, dasatinib given immediately following CAR-T cell infusion greatly reduced the death of mice from deadly CRS. Moreover, dasatinib quickly and reversibly stopped the CAR-T cells. As a result, this approach offers a brief suppression of CAR-T cell function and allows for the recovery of CAR-T cell treatment when toxicities have decreased. In the future, CAR-T cell therapy will need to improve in this aspect to become a first-line treatment for both blood cancers and solid tumors. This improvement will require more novel strategies that can quickly stop CAR-T cell function and enable the recovery of CAR-T cell therapy after the toxicity has passed [182].

## 12. Application of Artificial Intelligence in Manufacturing CAR-T Cells

The ability of computer systems to carry out tasks normally associated with human intellect, such as language translation, visual perception, learning, and decision making, is known as artificial intelligence (AI). AI in healthcare is the use of machine learning models to find insights and analyze medical data to improve patient experiences and health outcomes. AI has gained popularity in medical sciences in recent years because of its ability to analyze massive amount of data and problem-solving capabilities [183]. In particular, the use of AI has much potential and might eventually improve the production of CAR-T cells. Although having significant outcomes in cancer treatment, CAR-T cell therapy faces numerous limitations, such as avoiding immune toxicity and rejection, finding suitable targets for different types of cancer, and improving the efficiency and survival of CAR-T cells. Integrating AI applications can help to overcome these limitations by providing new insights and tools to improve accuracy, reduce costs, streamline logistics, enhance manufacturing efficiency, and improve the evaluation of CAR-T cell therapy. Some of these applications include finding a suitable target, cell manufacturing, gene editing, clinical trials, etc. [9].

AI finds novel specific targets for CAR-T therapy by exploring large-scale proteomic, transcriptomic, and genomic data from normal and cancer cells. For instance, a recent study employed AI to identify new targets for CAR-T therapy against a blood cancer know as acute myeloid leukemia that encountered challenges with conventional CAR-T cells [184,185]. AI assists in optimizing and standardizing the manufacturing process of CAR-T cells by managing and monitoring the quantity and quality of cells. AI, for example, can assist in automating the procedures of cell culture, purification, and expansion, in addition to detecting and eliminating defects or impurities in the cells [9]. Moreover, AI facilitates the gene-editing process of CAR-T cell by optimizing the development and delivery of CRISPR-Cas9, a genome editing tool that allows for the DNA modification of CAR-T cells. Immunological checkpoint molecules that inhibit the immune response, like PD-1, can be removed by using CRISPR-Cas9 to increase the anti-tumor effects of CAR-T cells. It can also be used to eliminate the TCR and MHC molecules to produce off-the-shelf or universal CAR-T cells that can be produced from healthy donors and prevent immunological rejection [186]. The clinical trials of CAR-T cell treatment can be designed and executed with the help of artificial intelligence by determining the ideal dose, schedule, and combination therapies, as well as managing and predicting the risk factors and outcomes of the therapy. AI analyzes patient data, including imaging, biomarkers, and symptoms, in order to determine the toxicity and response of CAR-T therapy and to offer personalized treatments [9].

Despite the integration of AI systems into current production and workflows, the validation of AI-based decisions still needs to be evaluated. The validation of AI models and data in the clinical settings and the ethical, legal, and societal implications of employing AI and gene editing in human cells are some of the challenges and limitations that must be overcome. The integration of AI in CAR-T cell treatment has the potential to accelerate further advancements in the field and increase the impact of this promising treatment for a variety of cancer types.

## 13. Conclusions and Future Prospects

CAR-T cell therapy has revolutionized the treatment of B cell hematological malignancies, but it also faces many challenges that limit its effectiveness and safety in solid tumors. Given the significant activity in the clinical settings of prostate, breast, renal, ovarian, colorectal, pancreatic, and thoracic cancer, the field is expected to overcome the challenges in a relatively short time, thus fulfilling the potential of CAR-T treatment for the curing of patients with aggressive forms of cancer, in addition to hematological malignancies. The field of CAR-T cell therapy is constantly evolving and new strategies and solutions are emerging that could improve the potential of this promising immunotherapy in the future. We discussed some of the most recent and promising treatment options of CAR-T cell therapy, such as the following.

The combination of CAR-T cell treatment and traditional immunotherapies has enormous promise for improving cancer outcomes and promoting personalized medicine. The future of CAR-T cell treatment is expected to be associated with its advancement as a combination therapy. Moreover, combination therapies, together with continuing innovation to reduce CAR-T toxicity and improve the efficiency of CAR-T cells, promise to bring out the best in each treatment, respectively. Thus, combination medicines may pave the way for safer and efficient medications, giving hope to cancer patients who have not responded to conventional therapies.

CRISPR-Cas9 offers a powerful gene-editing tool to engineer CAR-T cells in various ways, such as modifying the CAR structure, reducing the CAR immunogenicity, introducing CAR off-switches, improving CAR-T cell activity, and reducing the side effects. CRISPR-Cas9 holds promising applications for producing next-generation universal allogeneic CAR-T cell products that can be used for any patient and any tumor type, as well as the delivery of therapeutic genes to specific genomic locations and the targeting of negative regulators of T cell function. However, this approach still faces many challenges and limitations, including Cas9 protein immunogenicity, double-strand breaks, and off-target mutations, that need to be overcome before it can be translated into clinical practice. Researchers have developed multiplex prime editing, another gene editing technology to address the limitations associated with the CRISPR-Cas9 system. Multiplex prime editing has the potential to introduce precise alterations in various genomic regions without creating double-strand breaks and off-target mutations. This approach of multiplex prime editing could be used to insert therapeutic genes or fix mutations in CAR-T cells, broadening the usefulness and scope of this innovative immunotherapy. Thus, multiplex prime editing has the potential to significantly improve the efficiency and safety of CAR-T cell treatment for a variety of malignancies, especially solid tumors, in the near future.

The huge costs of the manufacturing process make CAR-T cell production a considerable problem. Furthermore, autologous CAR-T therapy is constrained to a made-to-order model, making economic manufacturing difficult to scale. Artificial intelligence (AI) technologies address the manufacturing challenges of CAR-T cell therapy by finding new targets for the optimal designing of CAR-T cells and monitoring the safety and effectiveness of CAR-T cell treatment in real time. A possible future treatment option is employing artificial intelligence in conjunction with combination therapies, multiplex prime editing, and Boolean-gated advanced-generation CARs, which could have the potential to completely transform the therapy of CAR-T cells.

## Figures and Tables

**Table 1:** Completed clinical trials of CAR-T therapy for cancer.

Trial ID	Trial Subjects	Phase	Intervention	Enrollments
NCT02659943	B Cell Lymphoma, NHLs	Phase1	Biological: Anti-CD19 CAR-T cells, Drug: fudarabine, cyclophosphamide	27
NCT02926833	Refractory DLBCL	Phase1, Phase 2	Biological: KTE-C19, Drug: atezolizumab, cyclophosphamide, fludarabine	37
NCT02215967	Myeloma	Phase1	Biological: anti-BCMA CAR-T cells, Drug: Fudarabine, cyclophosphamide	30
NCT00924326	B cell Lymphoma types	Phase1, Phase2	Biological: Anti-CD19, Drug: fludarabine, cyclophosphamide, aldesleukin, fludarabine, cyclophosphamide	43
NCT01865617	Recurrent Adult ALL, CLL, DLBCL, MCL, NHL, and Small Lymphocytic Lymphoma; Refractory ALL, Chronic Lymphocytic Leukemia, DLBCL, MCL, NHL, and Small Lymphocytic Lymphoma	Phase1, Phase2	Biological: Autologous Anti-CD19CAR-4-1BB-CD3zeta-EGFRt-expressing T lymphocytes	204
NCT03049449	Lymphoma, Large-Cell, Anaplastic, Enteropathy-Associated T cell Lymphoma, DLBCL, Extranodal NK-T cell Lymphoma	Phase1	Biological: anti-tumor necrosis factor receptor superfamily member 8 (CD 30) CAR-T cells, Drug: cyclophosphamide, fludarabine	26
NCT02348216	Refractory DLBCL, Relapsed DLBCL, TFL, PMBCL, HGBCL	Phase1, Phase2	Biological: axicabtagene ciloleucel, Drug: fludarabine, cyclophosphamide	307
NCT02761915	Relapsed or Refractory Neuroblastoma	Phase1	Procedure: leukapheresis, Drug: cyclophosphamide, fludarabine, Genetic: 1RG-CART	17
NCT01626495	B Cell Leukemia, B Cell Lymphoma	Phase1, Phase2	Biological: CART-19	73
NCT03338972	Refractory Plasma Cell Myeloma, Recurrent Plasma Cell Myeloma	Phase1	Biological: autologous anti-BCMA-CAR-expressing CD4+/CD8+ T-lymphocytes FCARH143, Procedure: leukapheresis Drug: fudarabine, cyclophosphamide	28
NCT01593696	Large-Cell Lymphoma, ALL, B Cell Lymphoma, Leukemia, Non-Hodgkin’s Lymphoma	Phase1	Biological: anti-CD19 CAR-T cells	53
NCT03289455	Recurrent Childhood Acute Lymphoblastic Leukemia, B Acute Lymphoblastic Leukemia, B cell Acute Lymphoblastic Leukemia, Refractory Childhood Acute Lymphoblastic Leukemia	Phase1, Phase2	Biological: AUTO3 (CD19/22) CAR-T cells	23
NCT03019055	B Cell Chronic Lymphocytic Leukemia, Non-Hodgkin’s Lymphoma, Small Lymphocytic Lymphoma	Phase1	Biological: CAR-20/19-T cells, CAR-20/19-T cells, CAR-20/19-T cells.	26
NCT02030847	Patients with refractory or relapsed B Cell ALL with no available curative treatment options	Phase2	Biological: CAR-T19	42
NCT02614066	Refractory/Relapsed B-precursor Acute Lymphoblastic Leukemia	Phase1, Phase2	Biological: brexucabtagene autoleucel, Drug: cyclophosphamide, fludarabine	125
NCT03958656	Myeloma, Multiple Myeloma	Phase1	Biological: anti-signaling lymphocytic activation molecule f7 (SLAMF7) CAR-T cells Drug: fludarabine, cyclophosphamide, fludarabine, rimiducid,	13
NCT03483103	NHL, DLBCL	Phase 2	Biological: lisocabtagene maraleucel	74
NCT03761056	B- ell Lymphoma	Phase2	Biological: axicabtagene ciloleucel, Drug: fludarabine, cyclophosphamide	42
NCT04456959	Precursor Cell Lymphoblastic Leukemia, Lymphoma		Drug: inotuzumab ozogamicin	28
NCT04030195	Non-Hodgkin’s Lymphoma Refractory, Non-Hodgkin’s Lymphoma, Chronic Lymphocytic Leukemia, Lymphoma, Non-Hodgkin’s, Leukemia, B cell Non-Hodgkin’s Lymphoma, B cell Chronic Lymphocytic Leukemia, Small Lymphocytic Lymphoma	Phase1, Phase2	Genetic: PBCAR20A, Drug: fludarabine, cyclophosphamide	18
NCT01454596	Brain Cancer, Malignant Glioma, Glioblastoma, Gliosarcoma	Phase1, Phase2	Biological: epidermal growth factor receptor (EGFRV)iii CAR-transduced PBL, Drug: fludarabine, aldesleukin, cyclophosphamide	18
NCT01460901	Neuroblastoma	Phase1	Biological: GD2 CAR-modified tri-virus-specific cytotoxic T cells	5
NCT02601313	Relapsed/Refractory Mantle Cell Lymphoma	Phase2	Biological: autoleucel, brexucabtagene Drug: cyclophosphamide, fludarabine, axicabtagene ciloleucel	105
NCT02650999	CD19+ DLBCL, Follicular Lymphomas, Mantle Cell Lymphomas	Phase1, Phase2	Drug: pembrolizumab	12

## Abbreviations

AI: artificial intelligence; ACT: adoptive cell therapy; APC: antigen-presenting cells; BCMA: B cell maturation antigen; BLG: Boolean logic-gated; CAR: chimeric antigen receptor; CEA: carcinoembryonic antigen; CCR: co-stimulatory receptor; CD: co-stimulatory domain; CEACAM7: carcinoembryonic antigen-related cell adhesion molecule; CRC: colorectal cancer; CRISPRs: clustered regularly interspaced short palindromic repeats; Dox: doxycycline; eTCR: engineered T cell receptor; EGFR; epidermal growth factor receptor; FDA: Food and Drug Administration; FLT3: FMS-like tyrosine kinase 3; gRNA: guide RNA; GPC3: glypican-3; GvHD: graft versus host disease; GPRC5D: G protein-coupled receptor, class C, group 5, member D; HER2: human epidermal growth factor receptor 2; HCC: hepatocellular carcinoma; HDR: homology-directed repair; HL: Hodgkin’s lymphoma; IFNγ: interferon gamma; IL: interleukin; ITD: internal tandem duplication; ITRs: inverted terminal repeats; IVT mRNA: in vitro transcribed mRNA; LILRB4: leukocyte immunoglobulin-like receptor-B4; LTRs: long terminal repeats; mAb: monoclonal antibody; MAS: macrophage activation syndrome; MHC: major histocompatibility complex; MM: multiple myeloma; Mo-MLV: Moloney murine leukemia virus; MSLN: mesothelin; MUC: mucin; NHEJ: non-homologous end joining; NHL: non-Hodgkin’s lymphoma; NK: natural killer cells; PD-1: programmed cell death protein 1; PSCA: prostate stem cell antigen; RR: response rate; R/R: relapsed/refractory; scFv: single-chain variable fragment; Siglec-6: sialic acid-binding immunoglobulin-like lectin 6; TAAs: tumor-associated antigens; TALENs: transcription activator-like effector nucleases; T-LBL; T cell lymphoblastic lymphoma; TAMs: tumor-associated macrophages; TCR: T cell receptor; TIL: tumor-infiltrating lymphocyte; TNF: tumor necrosis factor; TRAC: T cell receptor alpha chain; TLS: tumor lysis syndrome; Tregs: regulatory T cells; TRUCKs: T cells redirected for universal cytokine-mediated killing; TRUS: transrectal ultrasound scan; TGF: tumor growth factor; VL: variable light chain; VH: variable heavy chain; WBC: white blood cell; ZFNs: zinc fingers.

## References

[1] Cooper G.M., Hausman R.E. (2000). The development and causes of cancer. Cell A Mol. Approach.

[2] Bindea G., Mlecnik B., Angell H.K., Galon J. (2014). The immune landscape of human tumors: Implications for cancer immunotherapy. Oncoimmunology.

[3] Fridman W.H., Galon J., Dieu-Nosjean M.-C., Cremer I., Fisson S., Damotte D., Pagès F., Tartour E., Sautès-Fridman C. (2011). Immune infiltration in human cancer: Prognostic significance and disease control. Current Topics in Microbiology and Immunology.

[4] Varadé J., Magadán S., González-Fernández Á. (2021). Human immunology and immunotherapy: Main achievements and challenges. Cell. Mol. Immunol..

[5] Gupta S.L., Basu S., Soni V., Jaiswal R.K. (2022). Immunotherapy: An alternative promising therapeutic approach against cancers. Mol. Biol. Rep..

[6] Guedan S., Posey A.D., Shaw C., Wing A., Da T., Patel P.R., McGettigan S.E., Casado-Medrano V., Kawalekar O.U., Uribe-Herranz M. (2018). Enhancing CAR T cell persistence through ICOS and 4-1BB costimulation. JCI Insight.

[7] Westin J.R., Kersten M.J., Salles G., Abramson J.S., Schuster S.J., Locke F.L., Andreadis C. (2021). Efficacy and safety of CD19-directed CAR-T cell therapies in patients with relapsed/refractory aggressive B-cell lymphomas: Observations from the JULIET, ZUMA-1, and TRANSCEND trials. Am. J. Hematol..

[8] ImmPACT Bio (2023). ImmPACT Bio Announces FDA Clearance of IND for Novel Bispecific CAR to Treat Aggressive B-Cell Lymphoma. https://tdg.ucla.edu/immpact-bio-announces-fda-clearance-ind-novel-bispecific-car-treat-aggressive-b-cell-lymphoma#:~:text=(%E2%80%9CImmPACT%20Bio%E2%80%9D)%2C,a%20bispecific%20%E2%80%9COR%2DGate%E2%80%9D.

[9] Bäckel N., Hort S., Kis T., Nettleton D.F., Egan J.R., Jacobs J.J.L., Grunert D., Schmitt R.H. (2023). Elaborating the potential of Artificial Intelligence in automated CAR-T cell manufacturing. Front. Mol. Med..

[10] Carbone A. (2020). Cancer classification at the crossroads. Cancers.

[11] Kaur R., Bhardwaj A., Gupta S. (2023). Cancer treatment therapies: Traditional to modern approaches to combat cancers. Mol. Biol. Rep..

[12] Tan S., Li D., Zhu X. (2020). Cancer immunotherapy: Pros, cons and beyond. Biomed. Pharmacother..

[13] Waldman A.D., Fritz J.M., Lenardo M.J. (2020). A guide to cancer immunotherapy: From T cell basic science to clinical practice. Nat. Rev. Immunol..

[14] Klener P., Otahal P., Lateckova L. (2015). Immunotherapy approaches in cancer treatment. Curr. Pharm. Biotechnol..

[15] Goel G., Sun W. (2014). Cancer immunotherapy in clinical practice—The past, present, and future. Chin. J. Cancer.

[16] Topalian S.L., Hodi F.S., Brahmer J.R., Gettinger S.N., Smith D.C., McDermott D.F., Powderly J.D., Carvajal R.D., Sosman J.A., Atkins M.B. (2012). Safety, activity, and immune correlates of anti–PD-1 antibody in cancer. N. Engl. J. Med..

[17] Puhr H.C., Ilhan-Mutlu A. (2019). New emerging targets in cancer immunotherapy: The role of LAG3. ESMO Open.

[18] Qin S., Xu L., Yi M., Yu S., Wu K., Luo S. (2019). Novel immune checkpoint targets: Moving beyond PD-1 and CTLA-4. Mol. Cancer.

[19] Lee S., Margolin K. (2011). Cytokines in cancer immunotherapy. Cancers.

[20] Berraondo P., Sanmamed M.F., Ochoa M.C., Etxeberria I., Aznar M.A., Pérez-Gracia J.L., Rodriguez-Ruiz M.E., Ponz-Sarvise M., Castañón E., Melero I. (2019). Cytokines in clinical cancer immunotherapy. Br. J. Cancer.

[21] Southam C.M., Brunschwig A., Levin A.G., Dizon Q.S. (1966). Effect of leukocytes on transplantability of human cancer. Cancer.

[22] Weiden P.L., Flournoy N., Thomas E.D., Prentice R., Fefer A., Buckner C.D., Storb R. (1979). Antileukemic effect of graft-versus-host disease in human recipients of allogeneic-marrow grafts. N. Engl. J. Med..

[23] Rosenberg S.A., Yannelli J.R., Yang J.C., Topalian S.L., Schwartzentruber D.J., Weber J.S., Parkinson D.R., Seipp C.A., Einhorn J.H., White D.E. (1994). Treatment of patients with metastatic melanoma with autologous tumor-infiltrating lymphocytes and interleukin 2. JNCI J. Natl. Cancer Inst..

[24] Perica K., Varela J.C., Oelke M., Schneck J. (2015). Adoptive T cell immunotherapy for cancer. Rambam Maimonides Med. J..

[25] Garrido F., Aptsiauri N., Doorduijn E.M., Lora A.M.G., Van Hall T. (2016). The urgent need to recover MHC class I in cancers for effective immunotherapy. Curr. Opin. Immunol..

[26] Perez C.R., De Palma M. (2019). Engineering dendritic cell vaccines to improve cancer immunotherapy. Nat. Commun..

[27] Zhang Y., Zhang Z. (2020). The history and advances in cancer immunotherapy: Understanding the characteristics of tumor-infiltrating immune cells and their therapeutic implications. Cell. Mol. Immunol..

[28] Kamiya T., Seow S.V., Wong D., Robinson M., Campana D. (2019). Blocking expression of inhibitory receptor NKG2A overcomes tumor resistance to NK cells. J. Clin. Investig..

[29] Ochoa M.C., Minute L., Rodriguez I., Garasa S., Perez-Ruiz E., Inogés S., Melero I., Berraondo P. (2017). Antibody-dependent cell cytotoxicity: Immunotherapy strategies enhancing effector NK cells. Immunol. Cell Biol..

[30] Moon D., Tae N., Park Y., Lee S.-W., Kim D.H. (2022). Development of bispecific antibody for cancer immunotherapy: Focus on T cell engaging antibody. Immune Netw..

[31] Fesnak A.D., June C.H., Levine B.L. (2016). Engineered T cells: The promise and challenges of cancer immunotherapy. Nat. Rev. Cancer.

[32] Cappell K.M., Kochenderfer J.N. (2023). Long-term outcomes following CAR T cell therapy: What we know so far. Nat. Rev. Clin. Oncol..

[33] Zmievskaya E., Valiullina A., Ganeeva I., Petukhov A., Rizvanov A., Bulatov E. (2021). Application of CAR-T cell therapy beyond oncology: Autoimmune diseases and viral infections. Biomedicines.

[34] Zhang G., Wang L., Cui H., Wang X., Zhang G., Ma J., Han H., He W., Wang W., Zhao Y. (2014). Anti-melanoma activity of T cells redirected with a TCR-like chimeric antigen receptor. Sci. Rep..

[35] Chailyan A., Marcatili P., Tramontano A. (2011). The association of heavy and light chain variable domains in antibodies: Implications for antigen specificity. FEBS J..

[36] Liu X., Jiang S., Fang C., Yang S., Olalere D., Pequignot E.C., Cogdill A.P., Li N., Ramones M., Granda B. (2015). Affinity-tuned ErbB2 or EGFR chimeric antigen receptor T cells exhibit an increased therapeutic index against tumors in mice. Cancer Res..

[37] Caruso H.G., Hurton L.V., Najjar A., Rushworth D., Ang S., Olivares S., Mi T., Switzer K., Singh H., Huls H. (2015). Tuning sensitivity of CAR to EGFR density limits recognition of normal tissue while maintaining potent antitumor activity. Cancer Res..

[38] Jensen M.C., Riddell S.R. (2015). Designing chimeric antigen receptors to effectively and safely target tumors. Curr. Opin. Immunol..

[39] Srivastava S., Riddell S.R. (2015). Engineering CAR-T cells: Design concepts. Trends Immunol..

[40] Guest R.D., Hawkins R.E., Kirillova N., Cheadle E.J., Arnold J., O’Neill A., Irlam J., Chester K.A., Kemshead J.T., Shaw D.M. (2005). The role of extracellular spacer regions in the optimal design of chimeric immune receptors: Evaluation of four different scFvs and antigens. J. Immunother..

[41] Almåsbak H., Walseng E., Kristian A., Myhre M.R., Suso E.M., A Munthe L., Andersen J.T., Wang M.Y., Kvalheim G., Gaudernack G. (2015). Inclusion of an IgG1-Fc spacer abrogates efficacy of CD19 CAR T cells in a xenograft mouse model. Gene Ther..

[42] Zhang T., Wu M.-R., Sentman C.L. (2012). An NKp30-based chimeric antigen receptor promotes T cell effector functions and antitumor efficacy in vivo. J. Immunol..

[43] Dotti G., Gottschalk S., Savoldo B., Brenner M.K. (2014). Design and development of therapies using chimeric antigen receptor-expressing T cells. Immunol. Rev..

[44] Heuser C., Hombach A., Lösch C., Manista K., Abken H. (2003). T-cell activation by recombinant immunoreceptors: Impact of the intracellular signalling domain on the stability of receptor expression and antigen-specific activation of grafted T cells. Gene Ther..

[45] Zhao Y., Wang Q.J., Yang S., Kochenderfer J.N., Zheng Z., Zhong X., Sadelain M., Eshhar Z., Rosenberg S.A., Morgan R.A. (2009). A herceptin-based chimeric antigen receptor with modified signaling domains leads to enhanced survival of transduced T lymphocytes and antitumor activity. J. Immunol..

[46] Bridgeman J.S., Hawkins R.E., Bagley S., Blaylock M., Holland M., Gilham D.E. (2010). The optimal antigen response of chimeric antigen receptors harboring the CD3ζ transmembrane domain is dependent upon incorporation of the receptor into the endogenous TCR/CD3 complex. J. Immunol..

[47] Finney H.M., Lawson A.D., Bebbington C.R., Weir A.N.C. (1998). Chimeric receptors providing both primary and costimulatory signaling in T cells from a single gene product. J. Immunol..

[48] Acuto O., Michel F. (2003). CD28-mediated co-stimulation: A quantitative support for TCR signalling. Nat. Rev. Immunol..

[49] Cai B., Guo M., Wang Y., Zhang Y., Yang J., Guo Y., Dai H., Yu C., Sun Q., Qiao J. (2016). Co-infusion of haplo-identical CD19-chimeric antigen receptor T cells and stem cells achieved full donor engraftment in refractory acute lymphoblastic leukemia. J. Hematol. Oncol..

[50] Hu Y., Sun J., Wu Z., Yu J., Cui Q., Pu C., Liang B., Luo Y., Shi J., Jin A. (2016). Predominant cerebral cytokine release syndrome in CD19-directed chimeric antigen receptor-modified T cell therapy. J. Hematol. Oncol..

[51] Long A.H., Haso W.M., Shern J.F., Wanhainen K.M., Murgai M., Ingaramo M., Smith J.P., Walker A.J., Kohler M.E., Venkateshwara V.R. (2015). 4-1BB costimulation ameliorates T cell exhaustion induced by tonic signaling of chimeric antigen receptors. Nat. Med..

[52] Marin V., Pizzitola I., Agostoni V., Attianese G.M.P.G., Finney H., Lawson A., Pule M., Rousseau R., Biondi A., Biagi E. (2010). Cytokine-induced killer cells for cell therapy of acute myeloid leukemia: Improvement of their immune activity by expression of CD33-specific chimeric receptors. Haematologica.

[53] Ramos C.A., Rouce R., Robertson C.S., Reyna A., Narala N., Vyas G., Mehta B., Zhang H., Dakhova O., Carrum G. (2018). In vivo fate and activity of second-versus third-generation CD19-specific CAR-T cells in B cell non-Hodgkin’s lymphomas. Mol. Ther..

[54] Chmielewski M., Abken H. (2015). TRUCKs: The fourth generation of CARs. Expert Opin. Biol. Ther..

[55] Kagoya Y., Tanaka S., Guo T., Anczurowski M., Wang C.-H., Saso K., Butler M.O., Minden M.D., Hirano N. (2018). A novel chimeric antigen receptor containing a JAK–STAT signaling domain mediates superior antitumor effects. Nat. Med..

[56] Miyamoto T., Razavi S., DeRose R., Inoue T. (2013). Synthesizing biomolecule-based Boolean logic gates. ACS Synth. Biol..

[57] Tousley A.M., Rotiroti M.C., Labanieh L., Rysavy L.W., Rietberg S.P., de la Serna E.L., Sotillo E., Weber E.W., Rietberg S.P., Dalton G.N. (2022). Coopting T cell proximal signaling molecules enables Boolean logic-gated CAR T cell control. bioRxiv.

[58] Savanur M.A., Weinstein-Marom H., Gross G. (2021). Implementing logic gates for safer immunotherapy of cancer. Front. Immunol..

[59] Dejenie T.A., Tiruneh G., Medhin M., Terefe G.D., Admasu F.T., Tesega W.W., Abebe E.C. (2022). Current updates on generations, approvals, and clinical trials of CAR T-cell therapy. Hum. Vaccines Immunother..

[60] Fedorov V.D., Themeli M., Sadelain M. (2013). PD-1–and CTLA-4–based inhibitory chimeric antigen receptors (iCARs) divert off-target immunotherapy responses. Sci. Transl. Med..

[61] Abou-El-Enein M., Elsallab M., Feldman S.A., Fesnak A.D., Heslop H.E., Marks P., Till B.G., Bauer G., Savoldo B. (2021). Scalable manufacturing of CAR T cells for cancer immunotherapy. Blood Cancer Discov..

[62] Wang X., Rivière I. (2016). Clinical manufacturing of CAR T cells: Foundation of a promising therapy. Mol. Ther.-Oncolyt..

[63] Wang X., Stefanski J., Borquez-Ojeda O., Qu J., Hack A., He Q. (2015). Comparison of CTS Dynabeadsc CD3/CD28, Miltenyi TransAct CD3/28 and ExpAct Beads for Large-Scale CAR T Cell Manufacturing.

[64] Poltorak M.P., Graef P., Tschulik C., Wagner M., Cletiu V., Dreher S., Borjan B., Fraessle S.P., Effenberger M., Turk M. (2020). Expamers: A new technology to control T cell activation. Sci. Rep..

[65] Brudno J.N., Somerville R.P., Shi V., Rose J.J., Halverson D.C., Fowler D.H., Gea-Banacloche J.C., Pavletic S.Z., Hickstein D.D., Lu T.L. (2016). Allogeneic T cells that express an anti-CD19 chimeric antigen receptor induce remissions of B-cell malignancies that progress after allogeneic hematopoietic stem-cell transplantation without causing graft-versus-host disease. J. Clin. Oncol..

[66] Malone R.W., Felgner P.L., Verma I.M. (1989). Cationic liposome-mediated RNA transfection. Proc. Natl. Acad. Sci. USA.

[67] Zhao Y., Zheng Z., Cohen C.J., Gattinoni L., Palmer D.C., Restifo N.P., Rosenberg S.A., Morgan R.A. (2006). High-efficiency transfection of primary human and mouse T lymphocytes using RNA electroporation. Mol. Ther..

[68] Yu S., Li A., Liu Q., Li T., Yuan X., Han X., Wu K. (2017). Chimeric antigen receptor T cells: A novel therapy for solid tumors. J. Hematol. Oncol..

[69] Curran K.J., A Seinstra B., Nikhamin Y., Yeh R., Usachenko Y., van Leeuwen D.G., Purdon T., Pegram H.J., Brentjens R.J. (2015). Enhancing antitumor efficacy of chimeric antigen receptor T cells through constitutive CD40L expression. Mol. Ther..

[70] Moffett H.F., Coon M.E., Radtke S., Stephan S.B., McKnight L., Lambert A., Stoddard B.L., Kiem H.P., Stephan M.T. (2017). Hit-and-run programming of therapeutic cytoreagents using mRNA nanocarriers. Nat. Commun..

[71] Piscopo N.J., Mueller K.P., Das A., Hematti P., Murphy W.L., Palecek S.P., Capitini C.M., Saha K. (2018). Bioengineering solutions for manufacturing challenges in CAR T cells. Biotechnol. J..

[72] Tumaini B., Lee D.W., Lin T., Castiello L., Stroncek D.F., Mackall C., Wayne A., Sabatino M. (2013). Simplified process for the production of anti–CD19-CAR–engineered T cells. Cytotherapy.

[73] Cullen B.R., Lomedico P.T., Ju G. (1984). Transcriptional interference in avian retroviruses—Implications for the promoter insertion model of leukaemogenesis. Nature.

[74] Jin Z., Maiti S., Huls H., Singh H., Olivares S., Mátés L., Izsvák Z., Ivics Z., A Lee D., E Champlin R. (2011). The hyperactive Sleeping Beauty transposase SB100X improves the genetic modification of T cells to express a chimeric antigen receptor. Gene Ther..

[75] Monjezi R., Miskey C., Gogishvili T., Schleef M., Schmeer M., Einsele H., Ivics Z., Hudecek M. (2017). Enhanced CAR T-cell engineering using non-viral Sleeping Beauty transposition from minicircle vectors. Leukemia.

[76] Arif T., Farooq A., Ahmad F.J., Akhtar M., Choudhery M.S. (2023). Prime editing: A potential treatment option for β-thalassemia. Cell Biol. Int..

[77] Qasim W., Zhan H., Samarasinghe S., Adams S., Amrolia P., Stafford S., Butler K., Rivat C., Wright G., Somana K. (2017). Molecular remission of infant B-ALL after infusion of universal TALEN gene-edited CAR T cells. Sci. Transl. Med..

[78] DeWitt M.A., Corn J.E., Carroll D. (2017). Genome editing via delivery of Cas9 ribonucleoprotein. Methods.

[79] Liang X., Potter J., Kumar S., Zou Y., Quintanilla R., Sridharan M., Carte J., Chen W., Roark N., Ranganathan S. (2015). Rapid and highly efficient mammalian cell engineering via Cas9 protein transfection. J. Biotechnol..

[80] Ren J., Zhao Y. (2017). Advancing chimeric antigen receptor T cell therapy with CRISPR/Cas9. Protein Cell.

[81] Zhang C., Liu J., Zhong J., Zhang X. (2017). Engineering CAR-T cells. Biomark. Res..

[82] Ganeeva I., Zmievskaya E., Valiullina A., Kudriaeva A., Miftakhova R., Rybalov A., Bulatov E. (2022). Recent Advances in the Development of Bioreactors for Manufacturing of Adoptive Cell Immunotherapies. Bioengineering.

[83] Campbell A., Brieva T., Raviv L., Rowley J., Niss K., Brandwein H., Oh S., Karnieli O. (2015). Concise review: Process development considerations for cell therapy. Stem Cells Transl. Med..

[84] Choudhery M.S., Badowski M., Muise A., Pierce J., Harris D.T. (2014). Cryopreservation of whole adipose tissue for future use in regenerative medicine. J. Surg. Res..

[85] Abraham-Miranda J., Menges M., Atkins R., Mattie M., Kanska J., Turner J., Hidalgo-Vargas M.J., Locke F.L. (2022). CAR-T manufactured from frozen PBMC yield efficient function with prolonged in vitro production. Front. Immunol..

[86] Jandová M., Stacey G.N., Lánská M., Gregor J., Rozsívalová P., Beková L., Ducháová Z.W., Belada D., Radocha J., Měřička P. (2023). Perspective: The Role of Cryopreservation Techniques in Manufacturing, Transport, and Storage of Car-T Therapy Products. Cryoletters.

[87] Choudhery M.S., Badowski M., Muise A., THarris D. (2013). Utility of cryopreserved umbilical cord tissue for regenerative medicine. Curr. Stem Cell Res. Ther..

[88] Choudhery M.S., Harris D.T. (2014). Cryopreservation can be used as an anti-aging strategy. Cytotherapy.

[89] Bojic S., Murray A., Bentley B.L., Spindler R., Pawlik P., Cordeiro J.L., Bauer R., de Magalhães J.P. (2021). Winter is coming: The future of cryopreservation. BMC Biol..

[90] Amini L., Silbert S.K., Maude S.L., Nastoupil L.J., Ramos C.A., Brentjens R.J., Sauter C.S., Shah N.N., Abou-El-Enein M. (2022). Preparing for CAR T cell therapy: Patient selection, bridging therapies and lymphodepletion. Nat. Rev. Clin. Oncol..

[91] Owen K., Ghaly R., Shohdy K.S., Thistlethwaite F. (2023). Lymphodepleting chemotherapy practices and effect on safety and efficacy outcomes in patients with solid tumours undergoing T cell receptor-engineered T cell (TCR-T) Therapy: A systematic review and meta-analysis. Cancer Immunol. Immunother..

[92] Li S., Wang X., Yuan Z., Liu L., Luo L., Li Y., Wu K., Liu J., Yang C., Li Z. (2021). Eradication of T-ALL cells by CD7-targeted universal CAR-T cells and initial test of ruxolitinib-based CRS management. Clin. Cancer Res..

[93] Feng J., Xu H., Cinquina A., Wu Z., Chen Q., Zhang P., Wang X., Shan H., Xu L., Zhang Q. (2021). Treatment of aggressive T cell lymphoblastic lymphoma/leukemia using anti-CD5 CAR T cells. Stem Cell Rev. Rep..

[94] Pinz K., Liu H., Golightly M., Jares A., Lan F., Zieve G.W., Hagag N., Schuster M., E Firor A., Jiang X. (2016). Preclinical targeting of human T-cell malignancies using CD4-specific chimeric antigen receptor (CAR)-engineered T cells. Leukemia.

[95] Salman H., Pinz K.G., Wada M., Shuai X., Yan L.E., Petrov J.C., Ma Y. (2019). Preclinical targeting of human acute myeloid leukemia using CD4-specific chimeric antigen receptor (CAR) T cells and NK cells. J. Cancer.

[96] Xie L., Ma L., Liu S., Chang L., Wen F. (2021). Chimeric antigen receptor T cells targeting CD7 in a child with high-risk T-cell acute lymphoblastic leukemia. Int. Immunopharmacol..

[97] Alcantara M., Tesio M., June C.H., Houot R. (2018). CAR T-cells for T-cell malignancies: Challenges in distinguishing between therapeutic, normal, and neoplastic T-cells. Leukemia.

[98] Maciocia P.M., Wawrzyniecka P.A., Maciocia N.C., Burley A., Karpanasamy T., Devereaux S., Hoekx M., O’Connor D., Leon T.E., Rapoz-D’Silva T. (2022). Anti-CCR9 chimeric antigen receptor T cells for T-cell acute lymphoblastic leukemia. Blood J. Am. Soc. Hematol..

[99] Fanale M.A., Horwitz S.M., Forero-Torres A., Bartlett N.L., Advani R.H., Pro B., Chen R.W., Davies A., Illidge T., Uttarwar M. (2018). Five-year outcomes for frontline brentuximab vedotin with CHP for CD30-expressing peripheral T-cell lymphomas. Blood J. Am. Soc. Hematol..

[100] Nguyen D.H., Ball E.D., Varki A. (2006). Myeloid precursors and acute myeloid leukemia cells express multiple CD33-related Siglecs. Exp. Hematol..

[101] Meyer J.-E., Loff S., Dietrich J., Spehr J., Jiménez G.J., von Bonin M., Ehninger G., Cartellieri M., Ehninger A. (2021). Evaluation of switch-mediated costimulation in trans on universal CAR-T cells (UniCAR) targeting CD123-positive AML. Oncoimmunology.

[102] Tambaro F.P., Singh H., Jones E., Rytting M., Mahadeo K.M., Thompson P., Daver N., DiNardo C., Kadia T., Garcia-Manero G. (2021). Autologous CD33-CAR-T cells for treatment of relapsed/refractory acute myelogenous leukemia. Leukemia.

[103] Jetani H., Garcia-Cadenas I., Nerreter T., Thomas S., Rydzek J., Meijide J.B., Bonig H., Herr W., Sierra J., Einsele H. (2018). CAR T-cells targeting FLT3 have potent activity against FLT3− ITD+ AML and act synergistically with the FLT3-inhibitor crenolanib. Leukemia.

[104] Wang J., Chen S., Xiao W., Li W., Wang L., Yang S., Wang W., Xu L., Liao S., Liu W. (2018). CAR-T cells targeting CLL-1 as an approach to treat acute myeloid leukemia. J. Hematol. Oncol..

[105] Riether C., Pabst T., Höpner S., Bacher U., Hinterbrandner M., Banz Y., Müller R., Manz M.G., Gharib W.H., Francisco D. (2020). Targeting CD70 with cusatuzumab eliminates acute myeloid leukemia stem cells in patients treated with hypomethylating agents. Nat. Med..

[106] Zhang X., Zhu L., Zhang H., Chen S., Xiao Y. (2022). CAR-T cell therapy in hematological malignancies: Current opportunities and challenges. Front. Immunol..

[107] Jetani H., Navarro-Bailón A., Maucher M., Frenz S., Verbruggen C., Yeguas A., Vidriales M.B., González M., Saborido J.R., Kraus S. (2021). Siglec-6 is a novel target for CAR T-cell therapy in acute myeloid leukemia. Blood J. Am. Soc. Hematol..

[108] Li C., Cao W., Que Y., Wang Q., Xiao Y., Gu C., Wang D., Wang J., Jiang L., Xu H. (2021). A phase I study of anti-BCMA CAR T cell therapy in relapsed/refractory multiple myeloma and plasma cell leukemia. Clin. Transl. Med..

[109] Sharma P., Kanapuru B., George B., Lin X., Xu Z., Bryan W.W., Pazdur R., Theoret M.R. (2022). FDA approval summary: Idecabtagene vicleucel for relapsed or refractory multiple myeloma. Clin. Cancer Res..

[110] Sun C., Mahendravada A., Ballard B., Kale B., Ramos C., West J., Maguire T., McKay K., Lichtman E., Tuchman S. (2019). Safety and efficacy of targeting CD138 with a chimeric antigen receptor for the treatment of multiple myeloma. Oncotarget.

[111] Van de Donk N.W.C.J., Janmaat M.L., Mutis T., Lammerts Van Bueren J.J., Ahmadi T., Sasser A.K., Lokhorst H.M., Parren P.W.H.I. (2016). Monoclonal antibodies targeting CD 38 in hematological malignancies and beyond. Immunol. Rev..

[112] Radhakrishnan S.V., Luetkens T., Scherer S.D., Davis P., Mause E.R.V., Olson M.L., Yousef S., Panse J., Abdiche Y., Li K.D. (2020). CD229 CAR T cells eliminate multiple myeloma and tumor propagating cells without fratricide. Nat. Commun..

[113] Kriegsmann K., Kriegsmann M., Cremer M., Schmitt M., Dreger P., Goldschmidt H., Müller-Tidow C., Hundemer M. (2019). Cell-based immunotherapy approaches for multiple myeloma. Br. J. Cancer.

[114] Gogishvili T., Danhof S., Prommersberger S., Rydzek J., Schreder M., Brede C., Einsele H., Hudecek M. (2017). SLAMF7-CAR T cells eliminate myeloma and confer selective fratricide of SLAMF7+ normal lymphocytes. Blood J. Am. Soc. Hematol..

[115] Smith E.L., Harrington K., Staehr M., Masakayan R., Jones J., Long T.J., Ng K.Y., Ghoddusi M., Purdon T.J., Wang X. (2019). GPRC5D is a target for the immunotherapy of multiple myeloma with rationally designed CAR T cells. Sci. Transl. Med..

[116] Wang C.-M., Wu Z.-Q., Wang Y., Guo Y.-L., Dai H.-R., Wang X.-H., Li X., Zhang Y.-J., Zhang W.-Y., Chen M.-X. (2017). Autologous T cells expressing CD30 chimeric antigen receptors for relapsed or refractory Hodgkin lymphoma: An open-label phase I trial. Clin. Cancer Res..

[117] Voorhees T.J., Zhao B., Oldan J., Hucks G., Khandani A., Dittus C., Smith J., Morrison J.K., Cheng C.J., Ivanova A. (2022). Pretherapy metabolic tumor volume is associated with response to CD30 CAR T cells in Hodgkin lymphoma. Blood Adv..

[118] Zhou Z., Tao C., Li J., Tang J.C.-O., Chan A.S.-C., Zhou Y. (2022). Chimeric antigen receptor T cells applied to solid tumors. Front. Immunol..

[119] Hillerdal V., Essand M. (2015). Chimeric antigen receptor-engineered T cells for the treatment of metastatic prostate cancer. BioDrugs.

[120] Slovin S.F., Wang X., Hullings M., Arauz G., Bartido S., Lewis J.S., Schöder H., Zanzonico P., Scher H.I., Sadelain M. (2013). Chimeric antigen receptor (CAR+) modified T cells targeting prostate specific membrane antigen (PSMA) in patients (pts) with castrate metastatic prostate cancer (CMPC). Am. Soc. Clin. Oncol..

[121] Weimin S., Abula A., Qianghong D., Wenguang W. (2020). Chimeric cytokine receptor enhancing PSMA-CAR-T cell-mediated prostate cancer regression. Cancer Biol. Ther..

[122] Junghans R.P., Ma Q., Rathore R., Gomes E.M., Bais A.J., Lo A.S., Abedi M., Davies R.A., Cabral H.J., Al-Homsi A.S. (2016). Phase I trial of anti-PSMA designer CAR-T cells in prostate cancer: Possible role for interacting interleukin 2-T cell pharmacodynamics as a determinant of clinical response. Prostate.

[123] Wolf P., Alzubi J., Gratzke C., Cathomen T. (2021). The potential of CAR T cell therapy for prostate cancer. Nat. Rev. Urol..

[124] Ghoussoub R.A., Dillon D.A., D’Aquila T., Rimm E.B., Fearon E.R., Rimm D.L. (1998). Expression of c-met is a strong independent prognostic factor in breast carcinoma. Cancer Interdiscip. Int. J. Am. Cancer Soc..

[125] Yang Y.-H., Liu J.-W., Lu C., Wei J.-F. (2022). CAR-T cell therapy for breast cancer: From basic research to clinical application. Int. J. Biol. Sci..

[126] Tchou J., Zhao Y., Levine B.L., Zhang P.J., Davis M.M., Melenhorst J.J., Kulikovskaya I., Brennan A.L., Liu X., Lacey S.F. (2017). Safety and efficacy of intratumoral injections of chimeric antigen receptor (CAR) T cells in metastatic breast cancer. Cancer Immunol. Res..

[127] Shi D., Shi Y., Kaseb A.O., Qi X., Zhang Y., Chi J., Lu Q., Gao H., Jiang H., Wang H. (2020). Chimeric antigen receptor-glypican-3 T-cell therapy for advanced hepatocellular carcinoma: Results of phase I trials. Clin. Cancer Res..

[128] Wang Y., Chen M., Wu Z., Tong C., Dai H., Guo Y., Liu Y., Huang J., Lv H., Luo C. (2018). CD133-directed CAR T cells for advanced metastasis malignancies: A phase I trial. Oncoimmunology.

[129] Guo J., Tang Q. (2021). Recent updates on chimeric antigen receptor T cell therapy for hepatocellular carcinoma. Cancer Gene Therapy.

[130] Kershaw M.H., Westwood J.A., Parker L.L., Wang G., Eshhar Z., Mavroukakis S.A., White D.E., Wunderlich J.R., Canevari S., Rogers-Freezer L. (2006). A phase I study on adoptive immunotherapy using gene-modified T cells for ovarian cancer. Clin. Cancer Res..

[131] Aithal A., Rauth S., Kshirsagar P., Shah A., Lakshmanan I., Junker W.M., Jain M., Ponnusamy M.P., Batra S.K. (2018). MUC16 as a novel target for cancer therapy. Expert Opin. Ther. Targets.

[132] Katz S.C., Burga R.A., McCormack E., Wang L.J., Mooring W., Point G.R., Khare P.D., Thorn M., Ma Q., Stainken B.F. (2015). Phase I hepatic immunotherapy for metastases study of intra-arterial chimeric antigen receptor–modified T-cell therapy for CEA+ liver metastases. Clin. Cancer Res..

[133] Katz S.C., E Moody A., Guha P., Hardaway J.C., Prince E., LaPorte J., Stancu M., E Slansky J., Jordan K.R., Schulick R.D. (2020). HITM-SURE: Hepatic immunotherapy for metastases phase Ib anti-CEA CAR-T study utilizing pressure enabled drug delivery. J. Immunother. Cancer.

[134] Zhang C., Wang Z., Yang Z., Wang M., Li S., Li Y., Zhang R., Xiong Z., Wei Z., Shen J. (2017). Phase I escalating-dose trial of CAR-T therapy targeting CEA+ metastatic colorectal cancers. Mol. Ther..

[135] Prenen H., Dekervel J., Hendlisz A., Anguille S., Ahmad A., Cerf E., Jacques-Hespel C., Gauthy E., Agaugué S., Gilham D.E. (2021). Updated data from the alloSHRINK phase 1 first-in-Human study evaluating CYAD-101, an innovative non-Gene-Edited allogeneic CAR-T, in metastatic colorectal cancer. J. Clin. Oncol..

[136] Argani P., Iacobuzio-Donahue C., Ryu B., Rosty C., Goggins M., E Wilentz R., Murugesan S.R., Leach S.D., Jaffee E., Yeo C.J. (2001). Mesothelin is overexpressed in the vast majority of ductal adenocarcinomas of the pancreas: Identification of a new pancreatic cancer marker by serial analysis of gene expression (SAGE). Clin. Cancer Res..

[137] Posey A.D., Schwab R.D., Boesteanu A.C., Steentoft C., Mandel U., Engels B., Stone J.D., Madsen T.D., Schreiber K., Haines K.M. (2016). Engineered CAR T cells targeting the cancer-associated Tn-glycoform of the membrane mucin MUC1 control adenocarcinoma. Immunity.

[138] Raj D., Nikolaidi M., Garces I., Lorizio D., Castro N.M., Caiafa S.G., Moore K., Brown N.F., Kocher H.M., Duan X. (2021). CEACAM7 is an effective target for CAR T-cell therapy of pancreatic ductal adenocarcinoma. Clin. Cancer Res..

[139] Patel U., Abernathy J., Savani B.N., Oluwole O., Sengsayadeth S., Dholaria B. (2022). CAR T cell therapy in solid tumors: A review of current clinical trials. EJHaem.

[140] Adusumilli P.S., Zauderer M.G., Rusch V.W., O’Cearbhaill R.E., Zhu A., Ngai D.A., Zhu A., Cheema W., Chintala N.K., Halton E. (2019). Abstract CT036: A phase I clinical trial of malignant pleural disease treated with regionally delivered autologous mesothelin-targeted CAR T cells: Safety and efficacy. Cancer Res..

[141] Burns I., Gwynne W.D., Suk Y., Custers S., Chaudhry I., Venugopal C., Singh S.K. (2022). The Road to CAR T-Cell Therapies for Pediatric CNS Tumors: Obstacles and New Avenues. Front. Oncol..

[142] Adusumilli P.S., Zauderer M.G., Rivière I., Solomon S.B., Rusch V.W., O’Cearbhaill R.E., Zhu A., Cheema W., Chintala N.K., Halton E. (2021). A phase I trial of regional mesothelin-targeted CAR T-cell therapy in patients with malignant pleural disease, in combination with the anti–PD-1 agent pembrolizumab. Cancer Discov..

[143] Cordoba S., Onuoha S., Thomas S., Pignataro D.S., Hough R., Ghorashian S., Vora A., Bonney D., Veys P., Rao K. (2021). CAR T cells with dual targeting of CD19 and CD22 in pediatric and young adult patients with relapsed or refractory B cell acute lymphoblastic leukemia: A phase 1 trial. Nat. Med..

[144] Jacobson C.A., Chavez J.C., Sehgal A.R., William B.M., Munoz J., Salles G., Munshi P.N., Casulo C., Maloney D.G., de Vos S. (2022). Axicabtagene ciloleucel in relapsed or refractory indolent non-Hodgkin lymphoma (ZUMA-5): A single-arm, multicentre, phase 2 trial. Lancet Oncol..

[145] Teoh P.J., Chng W.J. (2021). CAR T-cell therapy in multiple myeloma: More room for improvement. Blood Cancer J..

[146] Fowler N.H., Dickinson M., Dreyling M., Martinez-Lopez J., Kolstad A., Butler J., Ghosh M., Popplewell L., Chavez J.C., Bachy E. (2022). Tisagenlecleucel in adult relapsed or refractory follicular lymphoma: The phase 2 ELARA trial. Nat. Med..

[147] Neelapu S.S., Locke F.L., Bartlett N.L., Lekakis L.J., Miklos D.B., Jacobson C.A., Braunschweig I., Oluwole O.O., Siddiqi T., Lin Y. (2017). Axicabtagene ciloleucel CAR T-cell therapy in refractory large B-cell lymphoma. N. Engl. J. Med..

[148] Maude S.L., Laetsch T.W., Buechner J., Rives S., Boyer M., Bittencourt H., Bader P., Verneris M.R., Stefanski H.E., Myers G.D. (2018). Tisagenlecleucel in children and young adults with B-cell lymphoblastic leukemia. N. Engl. J. Med..

[149] Schuster S.J., Bishop M.R., Tam C.S., Waller E.K., Borchmann P., McGuirk J.P., Jäger U., Jaglowski S., Andreadis C., Westin J.R. (2019). Tisagenlecleucel in adult relapsed or refractory diffuse large B-cell lymphoma. N. Engl. J. Med..

[150] Jain P., Nastoupil L., Westin J., Lee H.J., Navsaria L., Steiner R.E., Ahmed S., Moghrabi O., Oriabure O., Chen W. (2021). Outcomes and management of patients with mantle cell lymphoma after progression on brexucabtagene autoleucel therapy. Br. J. Haematol..

[151] Abramson J.S., Palomba M.L., Gordon L.I., Lunning M.A., Wang M., Arnason J., Mehta A., Purev E., Maloney D.G., Andreadis C. (2020). Lisocabtagene maraleucel for patients with relapsed or refractory large B-cell lymphomas (TRANSCEND NHL 001): A multicentre seamless design study. Lancet.

[152] Pan J., Tan Y., Wang G., Deng B., Ling Z., Song W., Seery S., Zhang Y., Peng S., Xu J. (2021). Donor-derived CD7 chimeric antigen receptor T cells for T-cell acute lymphoblastic leukemia: First-in-human, phase I trial. J. Clin. Oncol..

[153] Kirtane K., Elmariah H., Chung C.H., Abate-Daga D. (2021). Adoptive cellular therapy in solid tumor malignancies: Review of the literature and challenges ahead. J. Immunother. Cancer.

[154] Green D.J., Pont M., Sather B.D., Cowan A.J., Turtle C.J., Till B.G., Nagengast A.M., Libby E.N., Becker P.S., Coffey D.G. (2018). Fully human Bcma targeted chimeric antigen receptor T cells administered in a defined composition demonstrate potency at low doses in advanced stage high risk multiple myeloma. Blood.

[155] Brown C.E., Alizadeh D., Starr R., Weng L., Wagner J.R., Naranjo A., Ostberg J.R., Blanchard M.S., Kilpatrick J., Simpson J. (2016). Regression of glioblastoma after chimeric antigen receptor T-cell therapy. N. Engl. J. Med..

[156] Rafiq S., Hackett C.S., Brentjens R.J. (2020). Engineering strategies to overcome the current roadblocks in CAR T cell therapy. Nat. Rev. Clin. Oncol..

[157] Sterner R.C., Sterner R.M. (2021). CAR-T cell therapy: Current limitations and potential strategies. Blood Cancer J..

[158] Hege K.M., Bergsland E.K., Fisher G.A., Nemunaitis J.J., Warren R.S., McArthur J.G., Lin A.A., Schlom J., June C.H., Sherwin S.A. (2017). Safety, tumor trafficking and immunogenicity of chimeric antigen receptor (CAR)-T cells specific for TAG-72 in colorectal cancer. J. Immunother. Cancer.

[159] Murad J.P., Kozlowska A.K., Lee H.J., Ramamurthy M., Chang W.-C., Yazaki P., Colcher D., Shively J., Cristea M., Forman S.J. (2018). Effective targeting of TAG72+ peritoneal ovarian tumors via regional delivery of CAR-engineered T cells. Front. Immunol..

[160] Slaney C.Y., Kershaw M.H., Darcy P.K. (2014). Trafficking of T cells into tumors. Cancer Res..

[161] Priceman S.J., Tilakawardane D., Jeang B., Aguilar B., Murad J.P., Park A.K., Chang W.-C., Ostberg J.R., Neman J., Jandial R. (2018). Regional delivery of chimeric antigen receptor–engineered T cells effectively targets HER2+ breast cancer metastasis to the brain. Clin. Cancer Res..

[162] Brown C.E., Aguilar B., Starr R., Yang X., Chang W.-C., Weng L., Chang B., Sarkissian A., Brito A., Sanchez J.F. (2018). Optimization of IL13Rα2-targeted chimeric antigen receptor T cells for improved anti-tumor efficacy against glioblastoma. Mol. Ther..

[163] Quail D.F., Joyce J.A. (2013). Microenvironmental regulation of tumor progression and metastasis. Nat. Med..

[164] Chong E.A., Melenhorst J.J., Lacey S.F., Ambrose D.E., Gonzalez V., Levine B.L., June C.H., Schuster S.J. (2017). PD-1 blockade modulates chimeric antigen receptor (CAR)–modified T cells: Refueling the CAR. Blood J. Am. Soc. Hematol..

[165] Krenciute G., Prinzing B.L., Yi Z., Wu M.-F., Liu H., Dotti G., Balyasnikova I.V., Gottschalk S. (2017). Transgenic expression of IL15 improves antiglioma activity of IL13Rα2-CAR T cells but results in antigen loss variants. Cancer Immunol. Res..

[166] Roex G., Timmers M., Wouters K., Campillo-Davo D., Flumens D., Schroyens W., Chu Y., Berneman Z.N., Lion E., Luo F. (2020). Safety and clinical efficacy of BCMA CAR-T-cell therapy in multiple myeloma. J. Hematol. Oncol..

[167] Frey N.V., Porter D.L. (2016). Cytokine release syndrome with novel therapeutics for acute lymphoblastic leukemia. Hematology.

[168] Neelapu S.S., Tummala S., Kebriaei P., Wierda W., Gutierrez C., Locke F.L., Komanduri K.V., Lin Y., Jain N., Daver N. (2018). Chimeric antigen receptor T-cell therapy—Assessment and management of toxicities. Nat. Rev. Clin. Oncol..

[169] Davila M.L., Riviere I., Wang X., Bartido S., Park J., Curran K., Chung S.S., Stefanski J., Borquez-Ojeda O., Olszewska M. (2014). Efficacy and toxicity management of 19–28z CAR T cell therapy in B cell acute lymphoblastic leukemia. Sci. Transl. Med..

[170] Yáñez L., Sánchez-Escamilla M., Perales M.-A. (2019). CAR T cell toxicity: Current management and future directions. Hemasphere.

[171] June C.H., O’Connor R.S., Kawalekar O.U., Ghassemi S., Milone M.C. (2018). CAR T cell immunotherapy for human cancer. Science.

[172] Moore T.J., Zhang H., Anderson G., Alexander G.C. (2018). Estimated costs of pivotal trials for novel therapeutic agents approved by the US Food and Drug Administration, 2015–2016. JAMA Intern. Med..

[173] Cliff E.R.S., Kelkar A.H., Russler-Germain D.A., Tessema F.A., Raymakers A.J., Feldman W.B., Kesselheim A.S. (2023). high cost of chimeric antigen receptor T-cells: Challenges and solutions. Am. Soc. Clin. Oncol. Educ. Book.

[174] Milone M.C., Bhoj V.G. (2018). The pharmacology of T cell therapies. Mol. Ther.-Methods Clin. Dev..

[175] Van Der Stegen S.J., Hamieh M., Sadelain M. (2015). The pharmacology of second-generation chimeric antigen receptors. Nat. Rev. Drug Discov..

[176] Ying Z., Huang X.F., Xiang X., Liu Y., Kang X., Song Y., Guo X., Liu H., Ding N., Zhang T. (2019). A safe and potent anti-CD19 CAR T cell therapy. Nat. Med..

[177] Salter A.I., Ivey R.G., Kennedy J.J., Voillet V., Rajan A., Alderman E.J., Voytovich U.J., Lin C., Sommermeyer D., Liu L. (2018). Phosphoproteomic analysis of chimeric antigen receptor signaling reveals kinetic and quantitative differences that affect cell function. Sci. Signal..

[178] Sommermeyer D., Hill T., Shamah S.M., I Salter A., Chen Y., Mohler K.M., Riddell S.R. (2017). Fully human CD19-specific chimeric antigen receptors for T-cell therapy. Leukemia.

[179] Locke F.L., Neelapu S.S., Bartlett N.L., Lekakis L.J., Jacobson C.A., Braunschweig I., Oluwole O.O., Siddiqi T., Lin Y., Timmerman J.M. (2017). Preliminary results of prophylactic tocilizumab after axicabtageneciloleucel (axi-cel; KTE-C19) treatment for patients with refractory, aggressive non-Hodgkin lymphoma (NHL). Blood.

[180] Sterner R.M., Sakemura R., Cox M.J., Yang N., Khadka R.H., Forsman C.L., Hansen M.J., Jin F., Ayasoufi K., Hefazi M. (2019). GM-CSF inhibition reduces cytokine release syndrome and neuroinflammation but enhances CAR-T cell function in xenografts. Blood J. Am. Soc. Hematol..

[181] Jones B.S., Lamb L.S., Goldman F., Di Stasi A. (2014). Improving the safety of cell therapy products by suicide gene transfer. Front. Pharmacol..

[182] Mestermann K., Giavridis T., Weber J., Rydzek J., Frenz S., Nerreter T., Mades A., Sadelain M., Einsele H., Hudecek M. (2019). The tyrosine kinase inhibitor dasatinib acts as a pharmacologic on/off switch for CAR T cells. Sci. Transl. Med..

[183] Galimova R.M., Buzaev I.V., Ramilevich K.A., Yuldybaev L.K., Shaykhulova A.F. (2019). Artificial intelligence-developments in medicine in the last two years. Chronic Dis. Transl. Med..

[184] Gottschlich A., Thomas M., Grünmeier R., Lesch S., Rohrbacher L., Igl V., Briukhovetska D., Benmebarek M.-R., Vick B., Dede S. (2023). Single-cell transcriptomic atlas-guided development of CAR-T cells for the treatment of acute myeloid leukemia. Nat. Biotechnol..

[185] Bedoya A.D., Futoma J., Clement M.E., Corey K., Brajer N., Lin A., Simons M.G., Gao M., Nichols M., Balu S. (2020). Machine learning for early detection of sepsis: An internal and temporal validation study. JAMIA Open.

[186] Razeghian E., Nasution M.K.M., Rahman H.S., Gardanova Z.R., Abdelbasset W.K., Aravindhan S., Bokov D.O., Suksatan W., Nakhaei P., Shariatzadeh S. (2021). A deep insight into CRISPR/Cas9 application in CAR-T cell-based tumor immunotherapies. Stem Cell Res. Ther..

